# Predictability of Mortality in Patients With Myocardial Injury After Noncardiac Surgery Based on Perioperative Factors via Machine Learning: Retrospective Study

**DOI:** 10.2196/32771

**Published:** 2021-10-14

**Authors:** Seo Jeong Shin, Jungchan Park, Seung-Hwa Lee, Kwangmo Yang, Rae Woong Park

**Affiliations:** 1 Department of Biomedical Sciences Ajou University Graduate School of Medicine Suwon Republic of Korea; 2 Department of Anesthesiology and Pain Medicine Samsung Medical Center Sungkyunkwan University School of Medicine Seoul Republic of Korea; 3 Rehabilitation & Prevention Center, Heart Vascular Stroke Institute Samsung Medical Center Sungkyunkwan University School of Medicine Seoul Republic of Korea; 4 Department of Biomedical Engineering Seoul National University College of Medicine Seoul Republic of Korea; 5 Center for Health Promotion Samsung Medical Center Sungkyunkwan University School of Medicine Seoul Republic of Korea; 6 Department of Biomedical Informatics Ajou University School of Medicine Suwon Republic of Korea

**Keywords:** myocardial injury after noncardiac surgery, high-sensitivity cardiac troponin, machine learning, extreme gradient boosting

## Abstract

**Background:**

Myocardial injury after noncardiac surgery (MINS) is associated with increased postoperative mortality, but the relevant perioperative factors that contribute to the mortality of patients with MINS have not been fully evaluated.

**Objective:**

To establish a comprehensive body of knowledge relating to patients with MINS, we researched the best performing predictive model based on machine learning algorithms.

**Methods:**

Using clinical data from 7629 patients with MINS from the clinical data warehouse, we evaluated 8 machine learning algorithms for accuracy, precision, recall, F1 score, area under the receiver operating characteristic (AUROC) curve, and area under the precision-recall curve to investigate the best model for predicting mortality. Feature importance and Shapley Additive Explanations values were analyzed to explain the role of each clinical factor in patients with MINS.

**Results:**

Extreme gradient boosting outperformed the other models. The model showed an AUROC of 0.923 (95% CI 0.916-0.930). The AUROC of the model did not decrease in the test data set (0.894, 95% CI 0.86-0.922; *P*=.06). Antiplatelet drugs prescription, elevated C-reactive protein level, and beta blocker prescription were associated with reduced 30-day mortality.

**Conclusions:**

Predicting the mortality of patients with MINS was shown to be feasible using machine learning. By analyzing the impact of predictors, markers that should be cautiously monitored by clinicians may be identified.

## Introduction

Myocardial injury after noncardiac surgery (MINS) is associated with cardiovascular events and fivefold increased postoperative mortality, affecting up to the first 2 years after surgery [1]. Recently, MINS is accepted as the leading cause of postoperative mortality [2,3]. Along with the increased risk of mortality, the prevalence is also high, reported to be above 20% [2,3]. Many previous studies have reported risk factors for the occurrence of MINS [4-7], but relatively less attention has been given to perioperative factors that are associated with mortality in patients who were diagnosed with MINS. We reported perioperative factors that affect mortality after MINS [8-11]. However, our previous studies evaluated variables independently and not in a comprehensive manner.

In this study, we trained and evaluated machine learning models by leveraging the risk factors of patients with MINS and aimed to find a model with the best performance. Furthermore, we validated the performance of the model with the test data set that was curated by the same method with the training data set. By quantifying and comparing the effect of each variable on the predictive performance of the model, we developed a mobile app that predicts mortality in patients with MINS. Our findings may benefit a comprehensive understanding of patient characteristics related to mortality in patients with MINS.

## Methods

The Institutional Review Board at Samsung Medical Center forwent the approval for this study and the necessity to obtain informed consent for access to the Samsung Medical Center Troponin in Noncardiac Operation (SMC-TINCO) registry (SMC 2019-08-048) and the test data set for validation (SMC 2021-03-187), considering that both data sets were curated in deidentified form.

### Study Population and Data Curation

Samsung Medical Center is a tertiary referral center with nearly 2000 beds and more than 49,000 cases of surgeries performed every year. Additionally, they provide the clinical data warehouse called “Darwin-C,” which allows any researcher in the institution to automatically extract the deidentified data from this electronic medical record archive system (Multimedia Appendix 1). Using the “Darwin-C” system, we generated the SMC-TINCO registry (KCT0004244) and used it in this study. The SMC-TINCO contains consecutive data of 43,019 patients who had at least one inspection of cTn-I before or within 30 days after noncardiac surgery from January 2010 to June 2019.

The medical history was summarized by reviewing the preoperative assessment sheet, and the names and meanings of 44 features in the data sets are listed in Multimedia Appendix 2. The death state of the clinical data warehouse is consistently validated and updated from the National Population Registry of the Korea National Statistical Office.

The routine cTn-I assay of SMC was institutionally updated to high-sensitivity cTn-T from July 2019. Based on this change, we generated a data set for testing the model. The data set consists of 6246 adult patients who had postoperative high-sensitivity cTn-T measured within 30 days after noncardiac surgery between July 2019 and January 2021.

### Definitions and Study End Points

MINS was defined as peak postoperative cTn elevation above the 99th percentile of the normal limit within 30 days after surgery, but those with evidence of nonischemic etiology such as sepsis, pulmonary embolus, atrial fibrillation, cardioversion, or chronic elevation were not regarded as MINS based on the recent diagnostic criteria [12]. High-risk surgery was identified based on the 2014 European Society of Cardiology/Anesthesiology guidelines [13].

The primary end point was the predictability of 30-day mortality of patients with MINS based on perioperative factors. For the secondary outcome, we also evaluated the predictability of 1-year mortality.

### Perioperative Management and cTn Measurements

According to the institutional guidelines, postoperative cTn measurement is not an institutional routine practice. It is performed selectively on patients with one or more of the following major cardiovascular risk factors: heart failure, history of ischemic heart disease, stroke including transient ischemic attack, chronic kidney disease, diabetes mellitus on insulin therapy, or high-risk surgery, but symptoms may be determined at the discretion of the clinician [13].

An immunoassay (Advia Centaur XP, Siemens Healthcare Diagnostics, Erlangen, Germany) with high sensitivity was used for cTn-I. The lower detection limit was 6 ng/L, and 40 ng/L of the 99th percentile was the reference upper limit, as reported by the manufacturer [14]. In the test data set, a high-sensitivity assay of cTn-T (Elecsys, Roche, Basel, Switzerland) was analyzed using cobas e801 (Roche). The 99th percentile reference upper limit for hs-cTn-T was 14 ng/L.

### Development of Prediction Models

To compare the performance of prediction models, we investigated the eight widely used machine learning algorithms: extreme gradient boosting (XGB), generalized boosted regression model (GBM), random forests (RF), support vector machines (SVM), classification and regression trees (CART), linear discriminant analysis (LDA), lasso/ridge/elastic net (GLMNET), and k-nearest neighbors (kNN). The hyperparameters of each model were optimized based on a grid search using the area under the receiver operating characteristic (AUROC). Fivefold cross-validation was used in the model development. We evaluated each model according to the accuracy, precision, recall, F1 score, AUROC, and area under the precision and recall curve (AUPRC) values (Multimedia Appendix 3). We validated the performance of the trained model using a new test data set.

Feature importance and Shapley Additive Explanations (SHAP) values were used to present the impact of each feature on the performance of the prediction model. SHA*P* values show the characteristic of deriving a marginal distribution and weighted average by fixing all variables except one and randomly predicting that one to determine its importance [15]. Features are sorted in descending order by which the model contributes to classifying the data. Each patient was represented by one dot on each variable line. The horizontal location of each dot indicated whether the effect of a variable was associated with a higher or lower probability of death. The area on the right indicates the point where SHA*P* value is greater than zero. Variable-specific SHA*P* values >0 indicate an increased risk of death.

### Statistical Analysis

Differences were compared using *t* tests and presented as means and SDs in two-group comparisons. Categorical features were presented as numbers with percentages and compared using chi-square or Fisher exact tests. Statistical analyses were performed using R 3.6.3 (R Foundation for Statistical Computing). All tests were two-tailed, and *P*<.05 was considered to indicate statistical significance.

## Results

### Patient Characteristics

**Figure 1:**
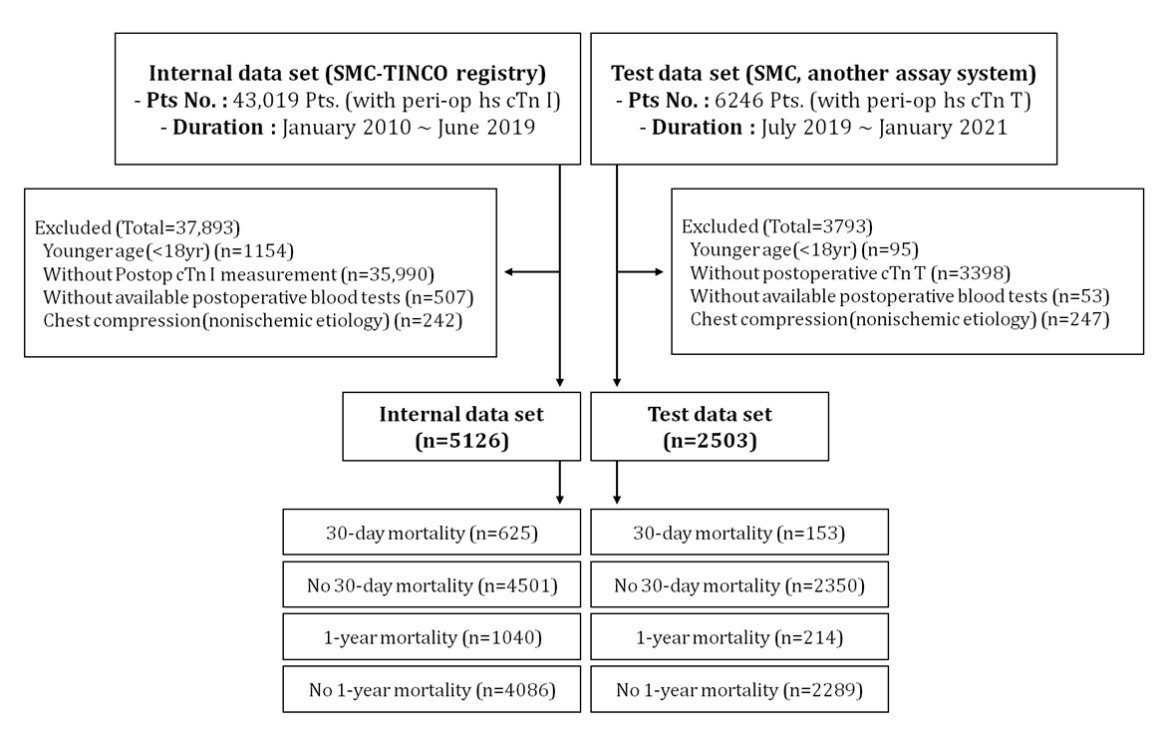
A flowchart of our retrospective study design. peri-op hs cTn I: perioperative high-sensitivity cTn-I; peri-op hs cTn T: perioperative high-sensitivity cTn-T; Pts: patients; SMN-TINCO: Samsung Medical Center Troponin in Noncardiac Operation.

In accordance with the definition of MINS, patients younger than 18 years were excluded from the data sets. Patients who did not have troponin measured after surgery or had abnormal levels and nonischemic etiology, such as chest compression, were also excluded (Figure 1). The baseline characteristics of the study patients with MINS are presented in Table 1. The age and gender of the patients in the training and test data sets showed a similar distribution (Multimedia Appendix 4), but the distribution of surgical types was slightly different. The number of patients in gynecology and urology in the test data set was increased, and other surgeries such as donor transplantation and bronchial dilation also varied (Multimedia Appendix 5). The type of surgery performed on patients in each data set and their mortality are presented in Multimedia Appendix 6.

**Table 1:** Baseline characteristics of patients with myocardial injury after noncardiac surgery according to 30-day mortality.

		Training data set				Test data set		
		No 30-day mortality (n=4501)	30-day mortality (n=625)	*P* value	No 30-day mortality (n=2350)		30-day mortality (n=153)	*P* value
Peak cardiac troponin level (ng/L), mean (SD)		2.3 (19.7)	7.2 (31.7)	<.001	0.1 (0.7)		0.6 (3.4)	.10
Male, n (%)		2673 (59.4)	394 (63.0)	.09	1526 (64.9)		89 (58.2)	.12
Age (years), mean (SD)		65.7 (13.8)	63.1 (14.2)	<.001	68.2 (12.8)		63.3 (13.6)	<.001
BMI, mean (SD)		23.7 (3.8)	22.9 (3.5)	<.001	24.0 (3.8)		22.7 (3.9)	<.001
Diabetes, n (%)		2480 (55.1)	363 (58.1)	.17	752 (32.0)		34 (22.2)	.02
Hypertension, n (%)		2994 (66.5)	378 (60.5)	.003	1209 (51.4)		55 (35.9)	<.001
Chronic kidney disease, n (%)		575 (12.8)	85 (13.6)	.61	429 (18.3)		21 (13.7)	.19
Dialysis, n (%)		231 (5.1)	47 (7.5)	.02	159 (6.8)		7 (4.6)	.38
Current smoking, n (%)		396 (8.8)	58 (9.3)	.75	157 (6.7)		16 (10.5)	.11
Current alcohol, n (%)		660 (14.7)	89 (14.2)	.83	260 (11.1)		15 (9.8)	.73
Coronary artery disease, n (%)		1059 (23.5)	111 (17.8)	.002	430 (18.3)		11 (7.2)	.001
**Previous disease**								
	Old myocardial infarction, n (%)	388 (8.6)	60 (9.6)	.46	215 (9.1)		12 (7.8)	.69
	History of coronary intervention, n (%)	530 (11.8)	36 (5.8)	<.001	304 (12.9)		10 (6.5)	.03
	History of coronary artery bypass graft, n (%)	120 (2.7)	17 (2.7)	>.99	66 (2.8)		4 (2.6)	>.99
	Heart failure, n (%)	174 (3.9)	14 (2.2)	.06	66 (2.8)		5 (3.3)	.94
	Stroke, n (%)	415 (9.2)	78 (12.5)	.01	253 (10.8)		17 (11.1)	>.99
	Atrial fibrillation, n (%)	356 (7.9)	55 (8.8)	.49	169 (7.2)		8 (5.2)	.45
	Arrhythmia, n (%)	453 (10.1)	63 (10.1)	>.99	229 (9.7)		12 (7.8)	.53
	Valvular heart disease, n (%)	95 (2.1)	8 (1.3)	.22	117 (5.0)		8 (5.2)	>.99
	Aortic disease, n (%)	136 (3.0)	14 (2.2)	.34	145 (6.2)		5 (3.3)	.20
	Peripheral arterial disease, n (%)	146 (3.2)	11 (1.8)	.06	91 (3.9)		7 (4.6)	.83
	Chronic pulmonary disease, n (%)	282 (6.3)	32 (5.1)	.30	206 (8.8)		9 (5.9)	.28
	Active cancer, n (%)	1751 (38.9)	262 (41.9)	.16	798 (34.0)		34 (22.2)	.004
	Charlson score, mean (SD)	3.2 (2.2)	3.8 (2.3)	<.001	2.1 (2.1)		1.6 (1.7)	<.001
**Operative variables**								
	ESC^a^/ESA^b^ surgical high risk, n (%)	1216 (27.0)	143 (22.9)	.03	524 (22.3)		38 (24.8)	.53
	Emergency operation, n (%)	1167 (25.9)	318 (50.9)	<.001	483 (20.6)		83 (54.2)	<.001
	General anesthesia, n (%)	3947 (87.7)	528 (84.5)	.03	2047 (87.1)		128 (83.7)	.27
	Operation duration (hours), mean (SD)	3.7 (2.8)	3.1 (2.8)	<.001	3.0 (2.2)		2.7 (2.3)	.12
	Packed red blood cell transfusion, n (%)	695 (15.4)	112 (17.9)	.13	0.5 (1.5)		1.1 (2.0)	<.001
**Postoperative in-hospital events**								
	Type I myocardial infarction, n (%)	104 (2.3)	16 (2.6)	.81	8 (0.3)		2 (1.3)	.24
	Coronary revascularization, n (%)	151 (3.4)	11 (1.8)	.04	28 (1.2)		2 (1.3)	>.99
	Percutaneous coronary intervention, n (%)	134 (3.0)	8 (1.3)	.02	27 (1.1)		2 (1.3)	>.99
	C-reactive protein level at discharge, mean (SD)	3.6 (4.0)	9.7 (8.8)	<.001	3.6 (4.4)		10.1 (8.7)	<.001
**Medication at discharge, n (%)**								
	Beta blocker	1031 (22.9)	13 (2.1)	<.001	294 (12.5)		5 (3.3)	.001
	Calcium channel blocker	1224 (27.2)	21 (3.4)	<.001	800 (34.0)		18 (11.8)	<.001
	Diltiazem	384 (8.5)	14 (2.2)	<.001	125 (5.3)		1 (0.7)	.02
	Stain	1165 (25.9)	12 (1.9)	<.001	933 (39.7)		9 (5.9)	<.001
	Metformin	497 (11.0)	26 (4.2)	<.001	474 (20.2)		7 (4.6)	<.001
	Insulin	1127 (25.0)	335 (53.6)	<.001	636 (27.1)		73 (47.7)	<.001
	Antiplatelet	1515 (33.7)	10 (1.6)	<.001	798 (34.0)		9 (5.9)	<.001
	Renin angiotensin aldosterone system inhibitor	1105 (24.6)	20 (3.2)	<.001	677 (28.8)		9 (5.9)	<.001
	Direct oral anticoagulant	211 (4.7)	3 (0.5)	<.001	6 (0.3)		0 (0.0)	>.99

^a^ESC: European Society of Cardiology.

^b^ESA: European Society of Anaesthesiology

### Development of a 30-Day Mortality Prediction Model

**Figure 2:**
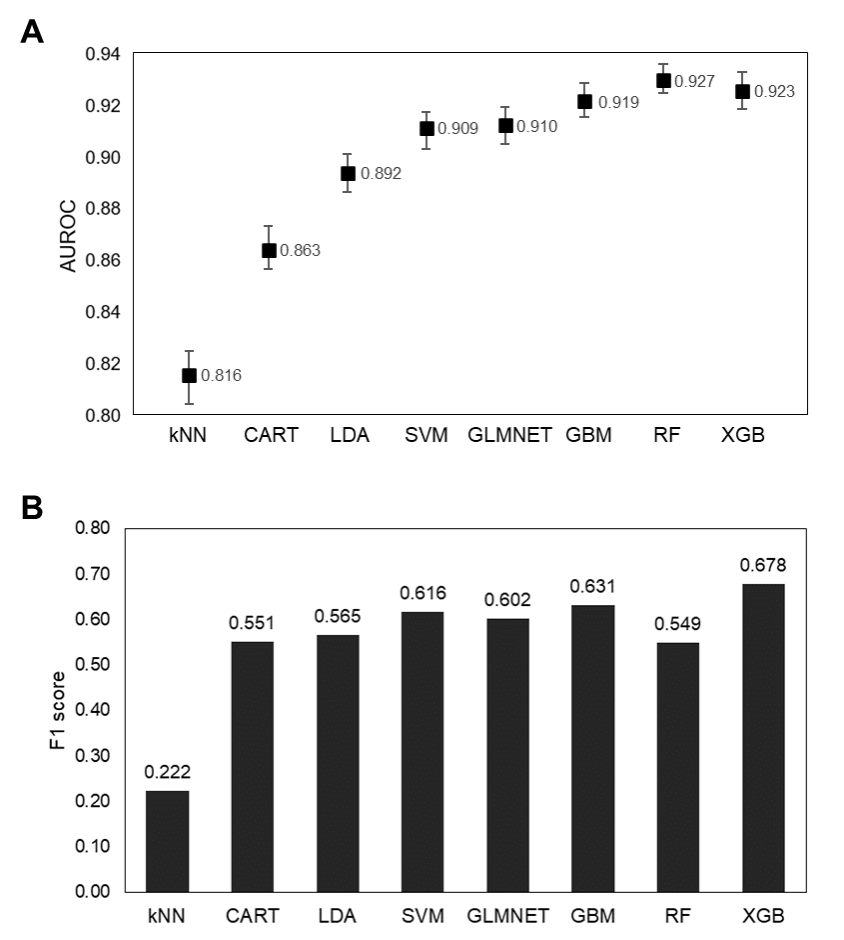
Performance comparison of each 30-day mortality prediction model with the range of (A) AUROC and (B) F1 score. AUROC: area under the receiver operating characteristic; CART: classification and regression trees; GBM: generalized boosted regression model; GLMNET: lasso/ridge/elastic net; kNN: k-nearest neighbors; LDA: linear discriminant analysis; RF: random forests; SVM: support vector machines; XGB: extreme gradient boosting.

The probability of developing a 30-day mortality prediction model was explored using 8 machine learning algorithms. The hyperparameters optimized using grid search are summarized in Multimedia Appendix 7. The performance of each model is displayed using AUROC and AUPRC plots (Multimedia Appendix 8) along with various indexes (Multimedia Appendix 9). The performance of the kNN, CART, LDA, SVM, GLMNET, and GBM models was lower than that of the RF and XGB models. The RF and XGB models showed comparable performances. The AUROC of the RF model (0.927) was higher than that of the XGB model (0.923) in the training phase. However, the AUPRC of the RF model (0.747) was lower than that of the XGB model (0.763). Additionally, the F1 score and balanced accuracy of the XGB model (0.678 and 0.784) were higher than those of the RF model (0.549 and 0.695). When the models were comprehensively evaluated, the XGB model was selected as the best performing model for predicting the 30-day mortality of patients with MINS (Figure 2).

### XGB 30-Day Mortality Prediction Model Interpretation

We tried to enable models to be actively accommodated by securing an interpretability and transparency. The importance of features in the XGB model is based on an algorithm that reduces based on the impurity index of the binary tree. The feature importance plot of the XGB 30-day mortality prediction model is shown in Multimedia Appendix 10. The top 5 features were C-reactive protein (CRP) level at discharge, antiplatelet prescription at discharge, peak cardiac troponin levels (ng/L), insulin prescription at discharge, and operation duration (hours).

**Figure 3:**
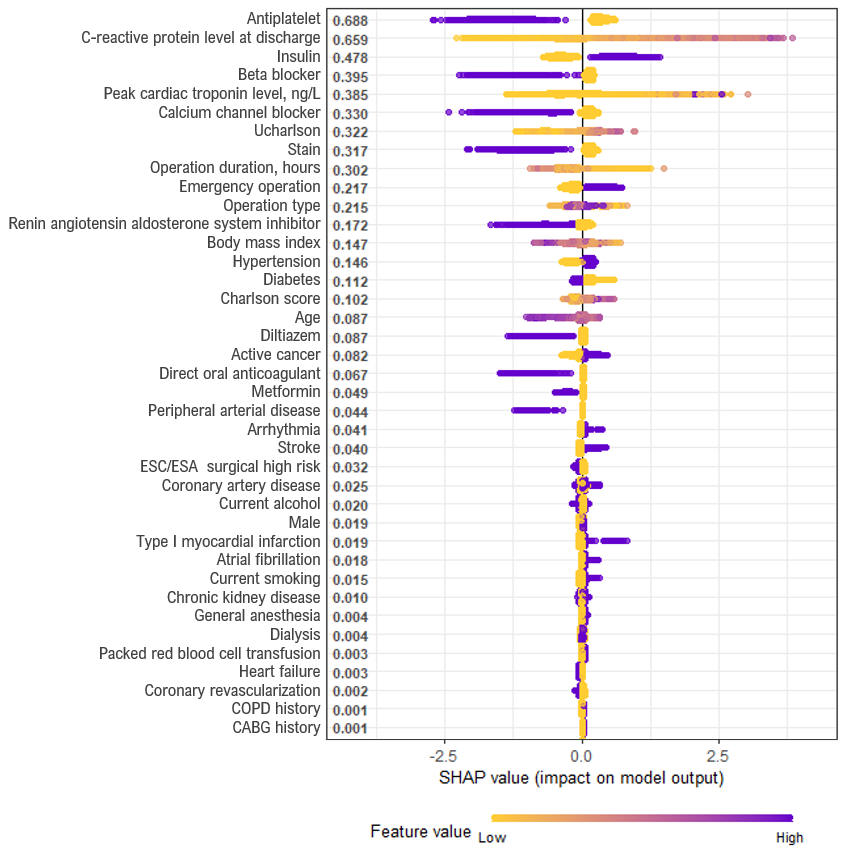
SHAP summary plot of 30-day mortality prediction extreme gradient boosting model. According to the SHA*P* values of each feature, antiplatelet prescription at discharge (ie, purple dots) was associated with a lower probability of death (ie, the left side of the vertical dotted line). Higher C-reactive protein levels at discharge (ie, purple dots) were associated with a higher probability of death (ie, the right side of the vertical dotted line). Insulin prescription at discharge (ie, purple dots) was associated with a higher probability of death (ie, the right side of the vertical dotted line). CABG: coronary artery bypass graft; COPD: chronic obstructive pulmonary disease; ESA: European Society of Anaesthesiology; ESC: European Society of Cardiology; SHAP: Shapley Additive Explanations.

The SHAP summary plot for the XGB models is shown in Figure 3. The XGB models determined that antiplatelet prescription at discharge was the most important variable, followed by CRP level at discharge, insulin prescription at discharge, beta blocker prescription at discharge, and peak cardiac troponin level (ng/L). According to the SHA*P* values of each feature, antiplatelet prescription at discharge was associated with a lower probability of death (left side of the vertical dotted lines). Higher CRP levels at discharge were associated with a higher probability of death. Insulin prescription at discharge was associated with higher probability of death. Additionally, a SHAP dependence plot was used to explain how a single feature affects the output of the XGB prediction model (Multimedia Appendix 11).

### Lightening the Model Using Feature Selection

By reducing the number of variables required to use predictive models, we tried to make the model more acceptable in clinical practice. We used the recursive feature elimination (RFE) method to explore the relation between the number of features and performance. According to the RFE method, the accuracy of the model is best when the top 28 variables were used. However, the performance of the model was almost the same as when the top 10 variables were used (Multimedia Appendix 12). To minimize the number of variables input into the model, we observed the changes in performance while reducing the number of variables to 28, 10, and 5.

#### Light Model With 28 Variables

The list of the top 28 predictor variables chosen by the RFE method is shown in Multimedia Appendix 12. When the top 28 variables were used to train the model, the performance of the XGB model had an accuracy of 0.926, AUPRC of 0.754, and F1 score of 0.652 (Multimedia Appendix 13). The AUROC was 0.925 (95% CI 0.919-0.931) in the training phase and 0.908 (95% CI 0.877-0.932) in the test phase. The AUROC of the model did not significantly decrease on the test data set (*P*=.22; Multimedia Appendix 14).

#### Light Model With 10 Variables

The top 10 variables used to train the XGB model were “crp_predc,” “insulin_dc,” “x_antiplt_dc,” “peaktro,” “ccb_dc,” “emergencyop,” “opduration,” “statin_dc,” “bb_dc,” and “optype.” The XGB model had an accuracy of 0.920, AUPRC of 0.708, and F1 score of 0.616 (Multimedia Appendix 13). The AUROC was 0.911 (95% CI 0.904-0.918) in the training phase and 0.904 (95% CI 0.874-0.93) in the test phase. The AUROC of the model did not significantly decrease on the test data set (*P*=.65; Multimedia Appendix 14).

#### Light Model With 10 Variables Chosen for Clinical Prediction

We made another model using 10 variables chosen for clinical prediction. Currently used treatments for MINS include dabigatran, a type of direct-acting oral coagulant [16], and potential treatments include antiplatelet agents and statins. We aimed to create a predictive model after excluding these drugs from the variables. The 10 chosen variables used were “crp_predc,” “insulin_dc,” “peaktro,” “ccb_dc,” “ccb_dc,” “emergencyop,” “opduration,” “bb_dc,” “optype,” “x_raas_dc,” and “metformin_dc.” The XGB model had an accuracy of 0.916, AUPRC of 0.672, and F1 score of 0.587 (Multimedia Appendix 13). The AUROC was 0.894 (95% CI 0.887-0.902) in the training phase and 0.895 (95% CI 0.867-0.923) in the test phase. The AUROC of the XGB model did not significantly decrease on the test data set (*P*=.99; Multimedia Appendix 14).

#### Light Model With 5 Variables

Multimedia Appendix 12 shows that the prediction accuracy decreased by approximately 1.9% when the model used 5 variables compared to when the model used 28 variables. For users who have only a small amount of information about patients with MINS, we made a lighter model by selecting 5 variables based on the RFE’s feature order. The top 5 variables used were “crp_predc,” “insulin_dc,” “x_antiplt_dc,” “peaktro,” and “ccb_dc.” The XGB model had an accuracy of 0.907, AUPRC of 0.640, and F1 score of 0.505 (Multimedia Appendix 13). The AUROC was 0.890 (95% CI 0.882-0.898) in the training phase and 0.885 (95% CI 0.856-0.915) in the test phase. The AUROC of the model did not significantly decrease on the test data set (*P*=.80; Multimedia Appendix 14).

### Development of a 1-Year Mortality Prediction Model

The AUROC of the 1-year mortality prediction XGB model was evaluated using the optimized hyperparameters eta=0.1, gamma=0, max tree depth=4, nround=100, colsample bytree=0.6, min child weight=1, and subsample=1. The AUROC of the model was 0.857 (95% CI 0.85-0.864) on the training data set and 0.794 (95% CI 0.756-0.826) on the test data set (Multimedia Appendix 15). The AUROC decreased on the test data set, and a statistically significant difference was observed compared to the AUROC of the training data set (*P*<.001). However, the prediction of the model is still valuable because the accuracy (0.95) on the test data set was above the no information rate (*P*=.001; Multimedia Appendix 16).

The feature importance plot of the 1-year mortality prediction model is shown in Multimedia Appendix 17. The top five features were the CRP level at discharge, peak cardiac troponin level (ng/L), operation duration (hours), antiplatelet prescription at discharge, and ucharlson score.

The SHAP summary plot for the models is shown in Multimedia Appendix 18. The XGB models determined that the CRP level at discharge was the most important variable, followed by the ucharlson score, antiplatelet prescription at discharge, insulin prescription at discharge, and operation duration (hours). According to the SHA*P* values of each feature, a higher CRP level at discharge and ucharlson score were associated with a higher probability of death. Antiplatelet prescription at discharge was associated with a lower probability of death, and insulin prescription at discharge was associated with a higher probability of death.

### Development of an App With 30-Day Mortality Prediction XGB Model

**Figure 4:**
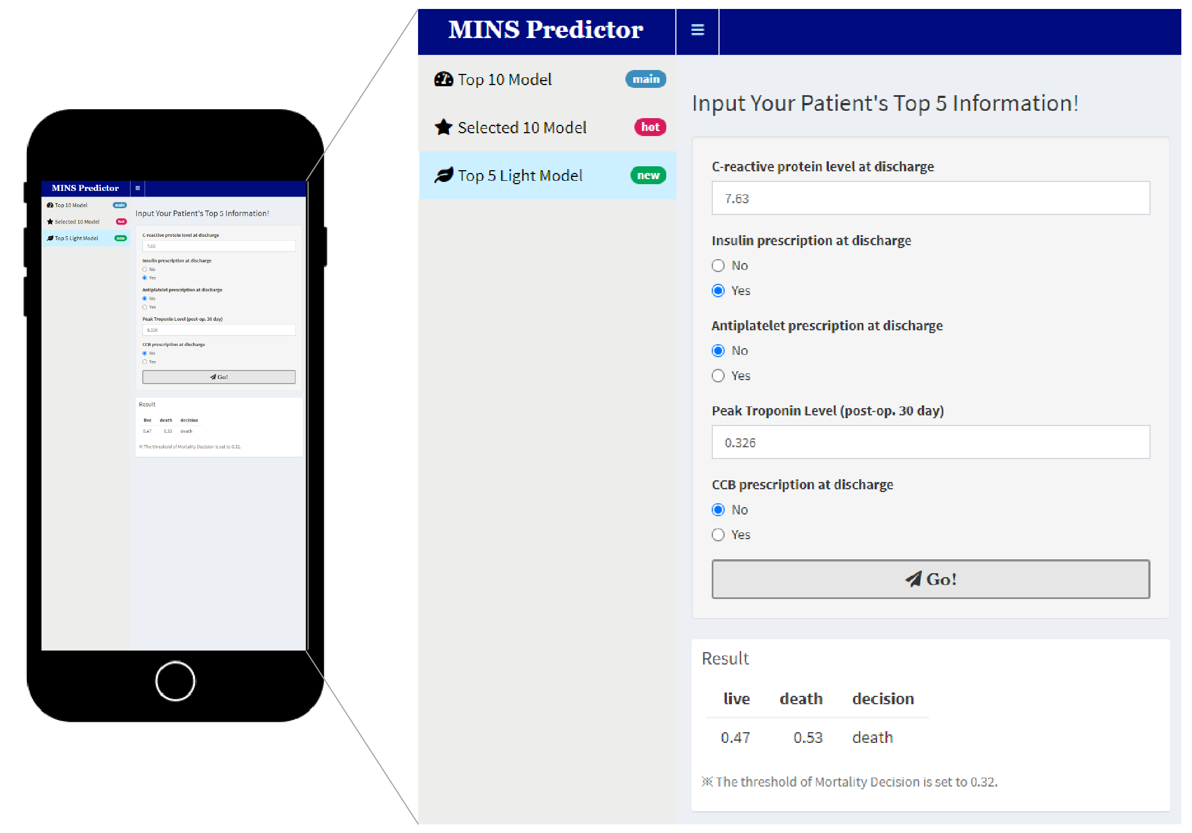
Internet app for predicting 30-day mortality of patients with MINS. CCB: calcium channel blockers; MINS: myocardial injury after noncardiac surgery.

The app, Leveraging R Shiny, was developed for practical use of the 30-day mortality prediction XGB model (Figure 4). Users can download the app for free via the public link [17]. Three versions of light models developed in this study are incorporated in the app: the top 10 features model, chosen 10 features model, and top 5 model. Each model was explored to find the optimal threshold for predicting patients at high risk of death. The optimized thresholds were applied to each model: 0.65 for the top 10 model; 0.53, chosen 10 model; and 0.68, top 5 model (Multimedia Appendix 19). Each user can choose a model type according to the type of variables that can be entered in a medical situation. A value for each variable corresponding to the target patient is entered and the Action button is pressed for probability output of the patient’s demise in 30 days. After adjusting certain variable values, clinicians can observe changes in mortality and apply them to treatment decisions.

## Discussion

In this observational cohort study, we demonstrated the predictability of mortality in patients with MINS based on perioperative variables using a machine learning method.

### Analysis of Model Performance Considering the Asymmetry of Data

To avoid overestimating the performance of the model, an imbalanced data set should be treated carefully when training a supervised classification machine learning model [18,19]. Along with accuracy, we wanted to interpret the performance of the model using indicators such as precision, recall, F1 score, AUPRC, and no information rate. In addition, for calibrating imbalanced data, four methods including oversampling, undersampling, both-sampling, and Random Over-Sampling Examples–sampling were carried out on the training data set, but the model’s performance was significantly reduced when the model was applied in the test data set; therefore, these methods were not accepted (data not shown).

### Comparison of 30-Day and 1-Year Preference Model Performance

We investigated why the 1-year prediction performance was lower than the 30-day prediction in this study. First, predicting the distant future is harder than predicting the near future. From a clinical perspective, although MINS has been reported to be associated with mortality up to 2 years after surgery, more clinical events that affect mortality are likely to take place as the duration of follow-up extends. Additionally, the observation period of the patients who made up the test data set (1.5 years) was shorter than that of the training data set (9.5 years). The observation period of the test data set may have been too short to reflect the characteristics of a patient who died within 1 year.

### Consideration of the SHAP Values of the Charlson Scores

We observed different relations to SHA*P* values between the original Charlson Comorbidity Index (CCI) scores and the updated CCI scores. The original CCI score shows a moderate proportional relationship with the SHA*P* value. However, the updated CCI score shows that the SHA*P* value increased rapidly in the low scores and was then maintained (Multimedia Appendix 20). It is assumed that the updated CCI score has changed the weights of cardiomyopathy, peripheral vascular disease, and cerebrovascular disease from 1 to 0.

### Clinical Implications

MINS is the most common medical complication directly related to mortality [13]. The rapid detection and appropriate management of MINS affects many patients at risk of mortality. The only treatment that was established in randomized trials was direct oral anticoagulants [16]. However, strengthening of cardiovascular drugs such as aspirin, statins, and few types of hypertension drugs have been reported to be linked to reduced mortality in patients with MINS [10,20]. Our results, show that the prescription of cardiovascular drugs such as antiplatelet agents, antihypertensive drugs, and statins at discharge are effective in predicting MINS mortality. The CRP level as a degree of inflammation is linked with the prognosis of coronary artery disease [21] and shows a strong impact on the model, which is consistent with previous studies. Therefore, our findings regarding the mortality of patients with MINS may be predicted based on perioperative variables, suggesting the possibility of reducing the mortality of patients with MINS by correction of perioperative variables.

We were able to reduce the number of variables to 5 with affordable loss in performance. Using only 5 variables, it is possible to predict the mortality of patients with MINS with 90.7% accuracy. A smaller number of variables in the prediction model indicates that it is highly likely to be used in other hospitals. Hence, we see this result as an important clinical implication.

### Limitations of the Study

Our study has a few limitations. First, model validation was performed using a test data set having a different time window from that of the data set used for training and internal validation. As a study using observational data collected in a single institution, our predictive models may have limited generalizability. Using a data set of patients with MINS visiting different institutions over the same period would allow for more appropriate external validation.

Second, our results might have been affected by selection bias and confounding factors. Postoperative hs-cTn measurements were not routine and optionally performed in patients with specific cardiovascular risks. Consequently, the possibility of selection bias may exist and should be considered if the user wants to apply the model in clinical practice.

Third, after confirming that mortality can be predicted using observational data, we created and released a mobile app for users. However, the predictive model developed in this study cannot be immediately used in routine clinical practice. We plan to conduct further research to measure the applicability of the model in clinical practice.

### Conclusions

We have confirmed that a 30-day mortality prediction model can be developed for patients with MINS using observational clinical data. The XGB algorithm outperformed the LDA, kNN, CART, SVM, GLMNET, RF, and GBM machine learning algorithms. To maximize the applicability of the prediction model in clinic settings, we observed that the number of variables that need to be input into the model can be reduced to 5 while preserving the performance of the model. For more robust evidence, a randomized clinical trial is required to address the variables explored in this study. However, this study is the first to report mortality predictability in patients with MINS using machine learning.

## Appendix

Multimedia Appendix 1 The clinical data warehouse of Samsung Medical Center, named DARWIN-C.

Multimedia Appendix 2 Name and meaning of features in original and test data sets.

Multimedia Appendix 3 Performance indicators for evaluating machine learning models.

Multimedia Appendix 4 Age distribution and sex of the patients in the two data sets.

Multimedia Appendix 5 Descriptive analysis of surgery type.

Multimedia Appendix 6 Surgery type and mortality.

Multimedia Appendix 7 The optimal parameters of the machine learning algorithm.

Multimedia Appendix 8 Area under the receiver operating characteristic and area under the precision and recall curve plots of each model in predicting 30-day mortality.

Multimedia Appendix 9 Performance indexes of machine learning models predicting 30-day mortality of patients with myocardial injury after noncardiac surgery.

Multimedia Appendix 10 Importance of features in the extreme gradient boosting 30-day mortality prediction model.

Multimedia Appendix 11 Shapley Additive Explanations dependence plots for top 10 features of 30-day mortality prediction model.

Multimedia Appendix 12 Recursive feature elimination graph based on accuracy.

Multimedia Appendix 13 Performance indexes of extreme gradient boosting models predicting 30-day mortality of patients with myocardial injury after noncardiac surgery with top 28, top 10, chosen 10, and top 5 variables.

Multimedia Appendix 14 Area under the receiver operating characteristic and area under the precision and recall curve plots of each model predicting 30-day mortality with top 28, top 10, chosen 10, and top 5 variables.

Multimedia Appendix 15 Area under the receiver operating characteristic and area under the precision and recall curve plots of the extreme gradient boosting model predicting 1-year mortality.

Multimedia Appendix 16 Performance indexes of extreme gradient boosting model predicting 1-year mortality of patients with myocardial injury after noncardiac surgery.

Multimedia Appendix 17 Importance of features in the extreme gradient boosting 1-year mortality prediction model.

Multimedia Appendix 18 Shapley Additive Explanations summary plot of 1-year mortality prediction extreme gradient boosting model.

Multimedia Appendix 19 Optimized threshold and final performance of models.

Multimedia Appendix 20 Comparison of the Shapley Additive Explanations value with the Charlson Comorbidity Index score and updated Charlson Comorbidity Index score.

## Abbreviations

AUPRC: area under the precision and recall curve
AUROC: area under the receiver operating characteristic
CART: classification and regression trees
CCI: Charlson Comorbidity Index
CRP: C-reactive protein
GBM: generalized boosted regression model
GLMNET: lasso/ridge/elastic net
kNN: k-nearest neighbors
LDA: linear discriminant analysis
MINS: myocardial injury after noncardiac surgery
RF: random forests
RFE: recursive feature elimination
SHAP: Shapley Additive Explanations
SMC-TINCO: Samsung Medical Center Troponin in Noncardiac Operation
SVM: support vector machines
XGB: extreme gradient boosting
