# Supplementary material for: Predictability of Mortality in Patients With Myocardial Injury After Noncardiac Surgery Based on Perioperative Factors via Machine Learning: Retrospective Study
Source: JMIR Med Inform. 2021 Oct 14;9(10):e32771. doi: 10.2196/32771 (PMC8554678; doi:10.2196/32771)

**Multimedia Appendix 1.** The Clinical Data Warehouse (CDW) of Samsung Medical Center, named DARWIN-C. It allows any researcher in the institution to automatically extract the de-identified data from this electronic medical record archive system. Screenshots of the login page (upper) and the system interface (lower).


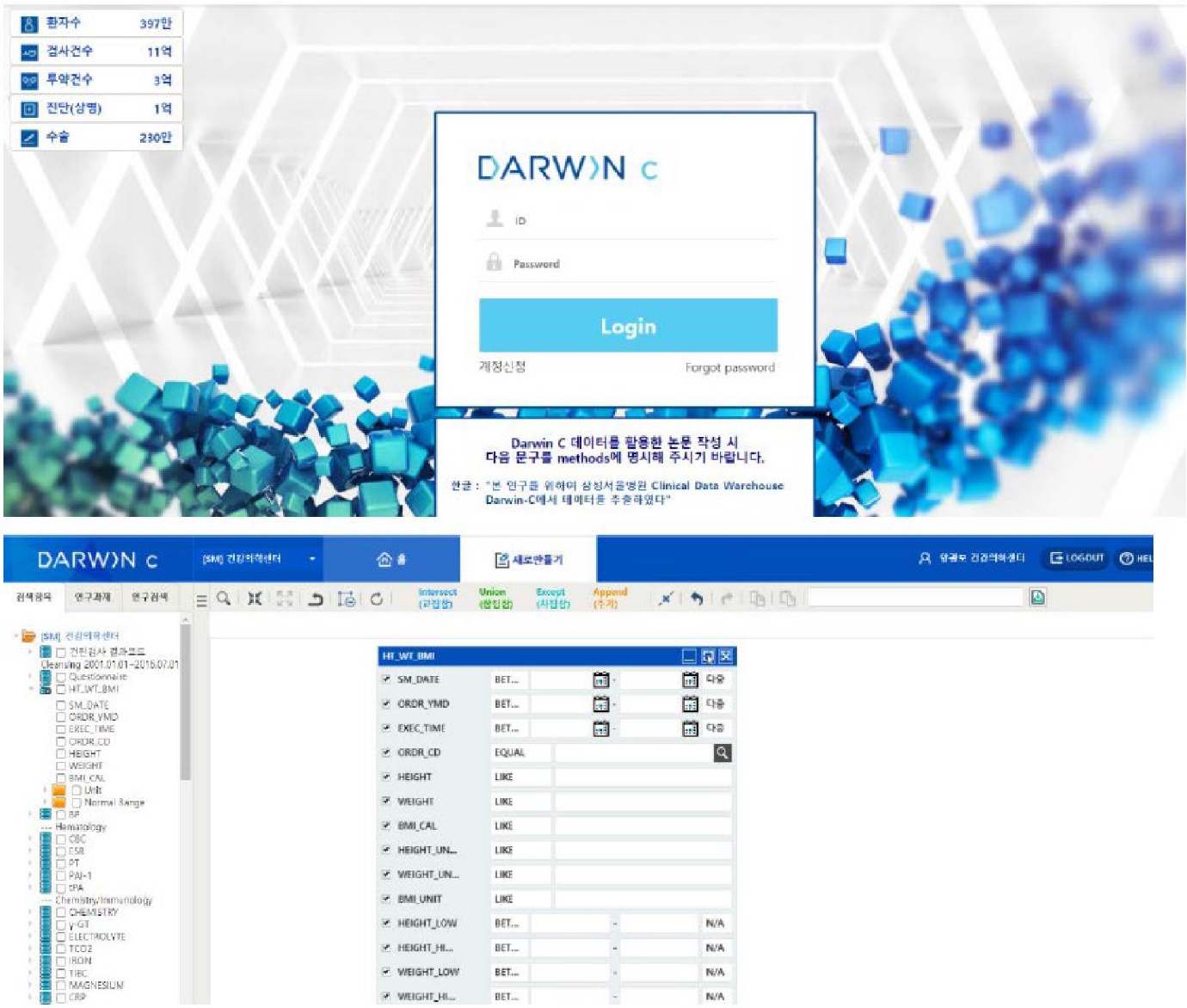

Supplement: Multimedia Appendix 1 [file medinform_v9i10e32771_app1.docx]
