# Supplementary material for: Predictability of Mortality in Patients With Myocardial Injury After Noncardiac Surgery Based on Perioperative Factors via Machine Learning: Retrospective Study
Source: JMIR Med Inform. 2021 Oct 14;9(10):e32771. doi: 10.2196/32771 (PMC8554678; doi:10.2196/32771)
Supplement: Multimedia Appendix 2 [file medinform_v9i10e32771_app2.docx]

**Multimedia Appendix 2.** Name and meaning of features in original and test datasets.

|  | **Original & Test dataset** | **Meaning** |
| --- | --- | --- |
| 1 | optype | Type of Surgery |
| 2 | sex | Sex |
| 3 | age | Age |
| 4 | bmi | Body Mass Index |
| 5 | peaktro | Peak Troponin Level (post operation 30 day) |
| 6 | crp_predc | C-reactive protein level at discharge |
| 7 | charlson | Charlson Comorbidity Index Score |
| 8 | ucharlson | Updated Charlson Comorbidity Index Score |
| 9 | oprisk | ESC/ESA surgical high risk |
| 10 | emergencyop | Emergency Operation |
| 11 | x_no_rbc_intraop | No. of Pack RBC Transfusion in operation |
| 12 | opduration | Operation Duration (hours) |
| 13 | alcohol | Current Alcohol |
| 14 | smoking | Current Smoking |
| 15 | ga | General Anesthesia |
| 16 | pci_inhos | Post Operation In-Hospital PCI |
| 17 | postmi | Myocardial Infarction (Post operation Angio. confirmed) |
| 18 | x_revas_inhos | Coronary revascularization (Post operation CABG, PCI) |
| 19 | x_activeca | Active Cancer (Diagnosis within 6 month) |
| 20 | crf | Chronic Renal Failure history |
| 21 | dialysis | Dialysis history |
| 22 | stroke | Stoke history |
| 23 | htn | Hypertension history |
| 24 | diabetes | Diabetes history |
| 25 | cad | Coronary Artery Disease history |
| 26 | omi | Myocardial Infarction history |
| 27 | pci | Percutaneous Intervention history |
| 28 | cabg | CABG history |
| 29 | hf | Heart Failure history |
| 30 | afib | Arterial Fibrillation history |
| 31 | arrhyth | Arrhythmia history |
| 32 | paod | Peripheral Arterial Occlusive Disease history |
| 33 | aorticdz | Aortic Disease history |
| 34 | valvedz | Valvular Heart Disease history |
| 35 | copd | COPD history |
| 36 | x_antiplt_dc | Antiplatelet prescription at discharge |
| 37 | x_doac_dc | DOAC prescription at discharge |
| 38 | statin_dc | Statin prescription at discharge |
| 39 | x_raas_dc | RASS prescription at discharge |
| 40 | bb_dc | Beta-blocker prescription at discharge |
| 41 | ccb_dc | CCBs prescription at discharge |
| 42 | dilv_dc | Diltiazem or Verapamil prescription at discharge |
| 43 | metformin_dc | Metformin prescription at discharge |
| 44 | insulin_dc | Insulin prescription at discharge |

^a^ESC: European Society of Cardiology, ^b^ESA: European Society of Anaesthesiology, ^c^RBC: Red Blood Cell, ^d^PCI: Percutaneous Coronary Intervention, ^e^CABG: Coronary Artery Bypass Grafting, ^f^COPD: Chronic Obstructive Pulmonary Disease, ^g^DOAC: Direct Oral Anticoagulants, ^h^RASS: Renin-angiotensin-aldosterone system, ^i^CCBs: Calcium Channel Blockers
