# Supplementary material for: Predictability of Mortality in Patients With Myocardial Injury After Noncardiac Surgery Based on Perioperative Factors via Machine Learning: Retrospective Study
Source: JMIR Med Inform. 2021 Oct 14;9(10):e32771. doi: 10.2196/32771 (PMC8554678; doi:10.2196/32771)
Supplement: Multimedia Appendix 3 [file medinform_v9i10e32771_app3.docx]

**Multimedia Appendix 3**. Performance indicators for evaluating machine learning models.

| **Indicator** | **Meaning** |
| --- | --- |
| Accuracy | Accordance ratio between predicted and actual value  (TP^a^ + TN^b^) / (TP^a^ + FP^c^ +FN^d^ + TN^b^) |
| Precision (positive predictive value) | Ratio of actual True of the value predicted as True TP^a^/ (TP^a^ + FP^c^) |
| Recall (sensitivity, true positive rate) | Ratio of value predicted as True of actual True TP^a^/ (TP^a^ + FN^d^) |
| F1 score | Harmonic average of Precision and Recall 2 × (recall × precision) / (recall + precision) |
| AUROC^e^ | The area under the receiver operating characteristic curve  (plotting TPR^f^ against FPR^g^) Higher AUROC^e^, close to 1 = better classifier |
| AUPRC^h^ | The area under the precision-recall curve (Plotting precision against recall) Higher AUPRC^h^, close to 1 = better classifier Advantage over AUROC^e^ when comparing the performance of models in an imbalanced dataset |

^a^TP: True Positive, ^b^TN: True Negative, ^c^FP: False Positive, ^d^FN: False Negative, ^e^AUROC: Area Under the Receiver Operating Characteristic, ^f^TPR: True Positive Rate, ^g^FPR: False Positive Rate, ^h^AUPRC: Area Under the Precision Recall Curve
