# Supplementary figures and images for: Predictability of Mortality in Patients With Myocardial Injury After Noncardiac Surgery Based on Perioperative Factors via Machine Learning: Retrospective Study
Source: JMIR Med Inform. 2021 Oct 14;9(10):e32771. doi: 10.2196/32771 (PMC8554678; doi:10.2196/32771)

**Multimedia Appendix 4.** Age distribution and sex of the patients in the two datasets.


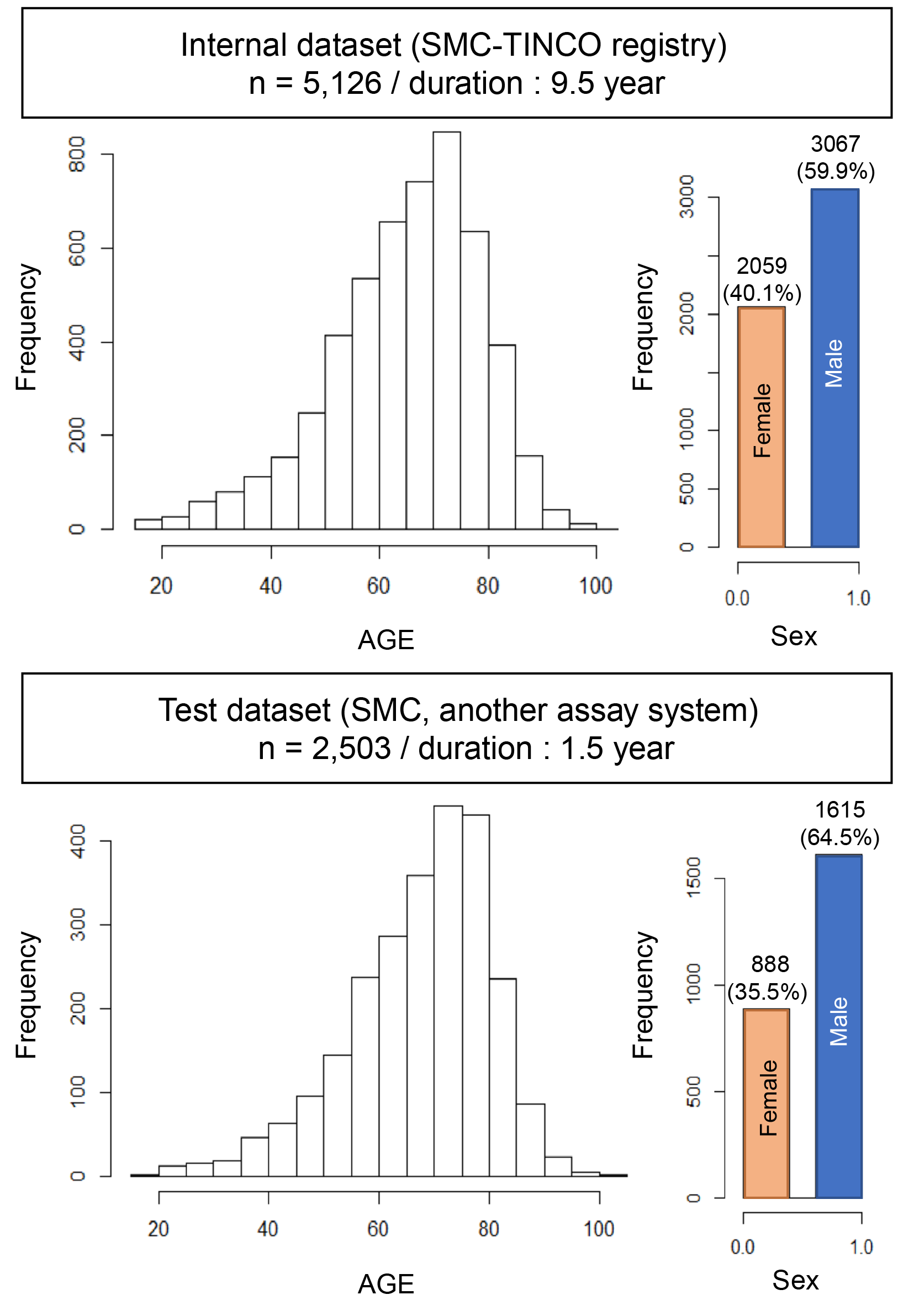

Supplement: Multimedia Appendix 4 [file medinform_v9i10e32771_app4.docx]

**Multimedia Appendix 10.** Importance of features in the XGB 30-day mortality prediction model.


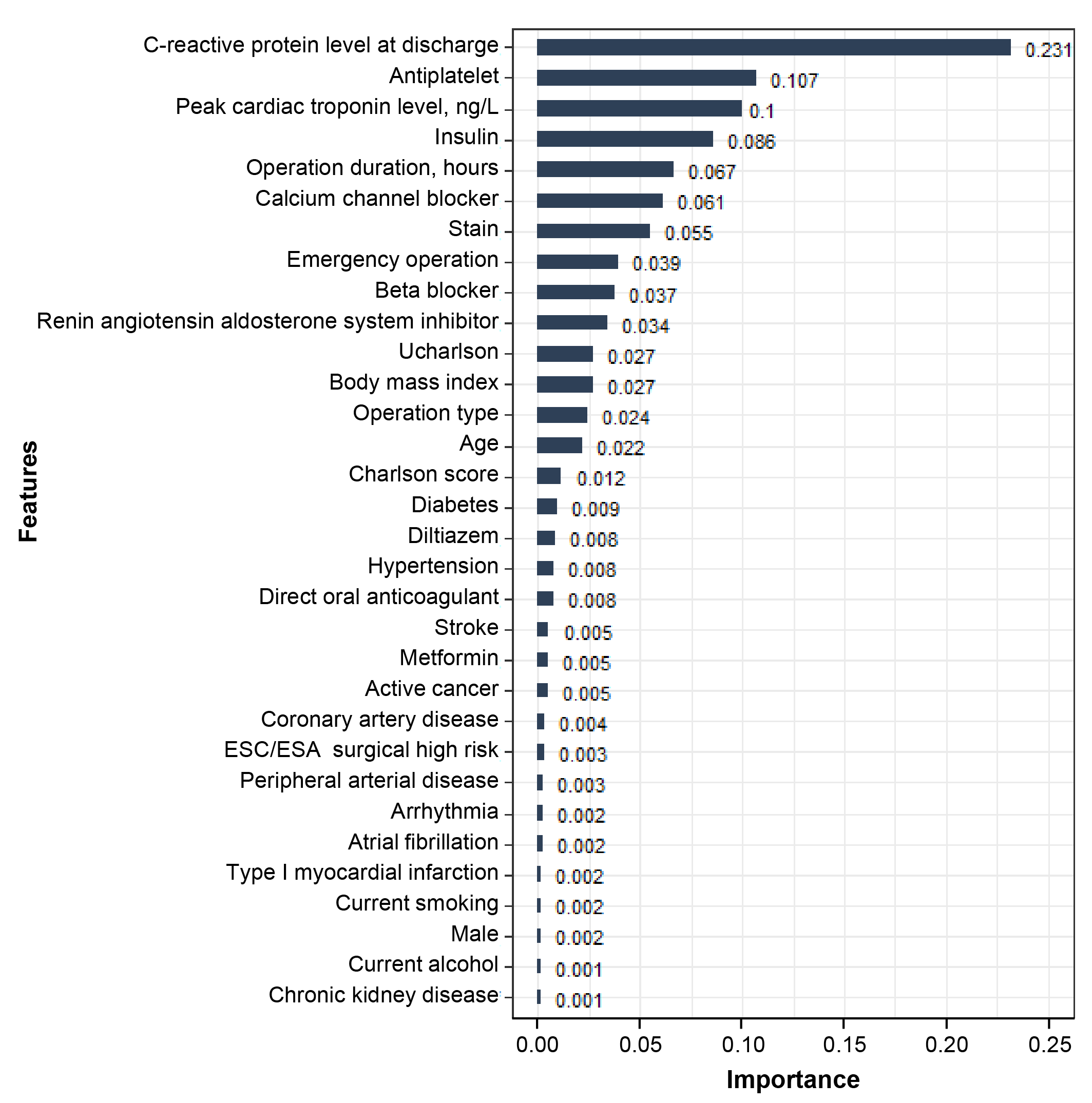

Supplement: Multimedia Appendix 10 [file medinform_v9i10e32771_app10.docx]

**Multimedia Appendix 15**. (a) AUROC and (b) AUPRC plots of XGB model predicting one-year mortality.


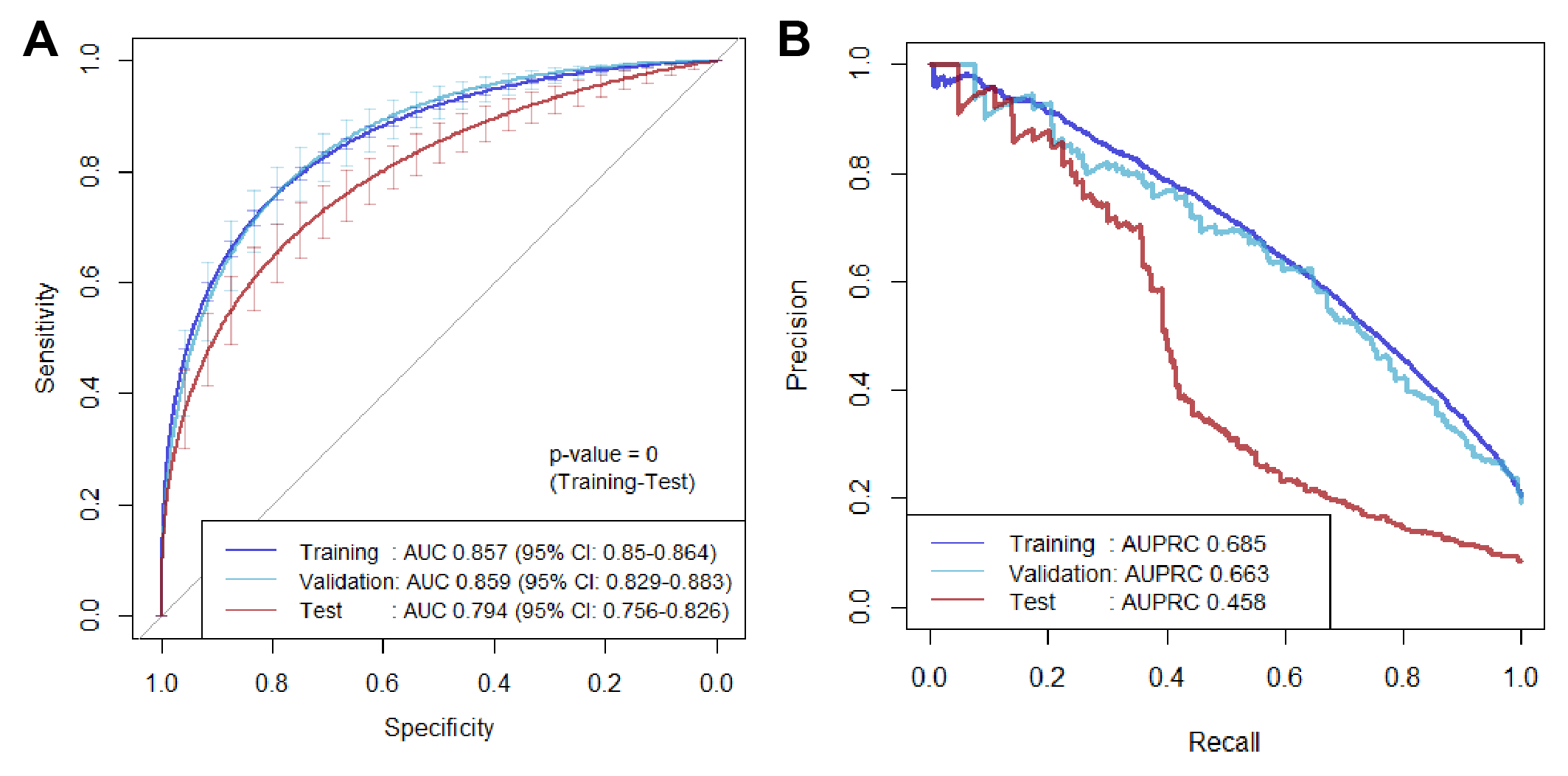

Supplement: Multimedia Appendix 15 [file medinform_v9i10e32771_app15.docx]

**Multimedia Appendix 17**. Importance of features in the XGB one-year mortality prediction model.


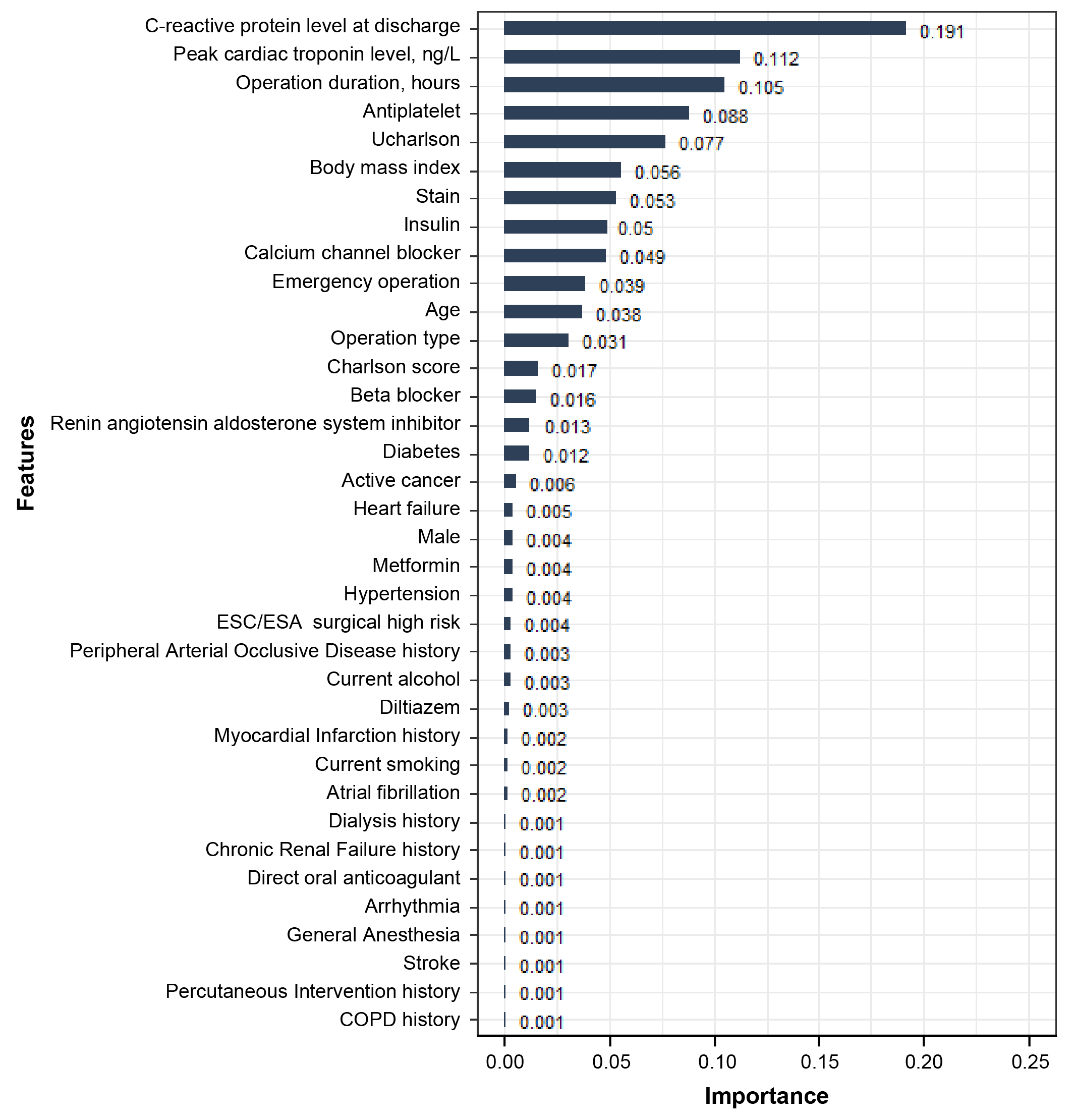

Supplement: Multimedia Appendix 17 [file medinform_v9i10e32771_app17.docx]

**Multimedia Appendix 18**. SHAP summary plot of one-year mortality prediction XGB model.


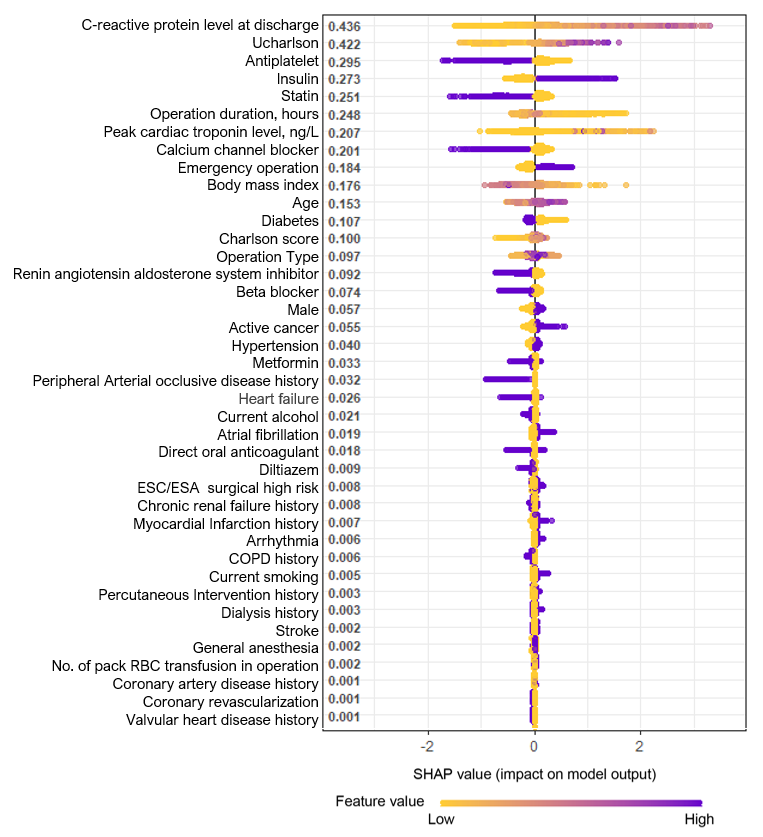

Supplement: Multimedia Appendix 18 [file medinform_v9i10e32771_app18.docx]
