# Supplementary material for: Predictability of Mortality in Patients With Myocardial Injury After Noncardiac Surgery Based on Perioperative Factors via Machine Learning: Retrospective Study
Source: JMIR Med Inform. 2021 Oct 14;9(10):e32771. doi: 10.2196/32771 (PMC8554678; doi:10.2196/32771)

**Multimedia Appendix 5.** Descriptive analysis of surgery type. The two datasets had different numbers of surgery types, especially in Gynecology, Urology, and Others.


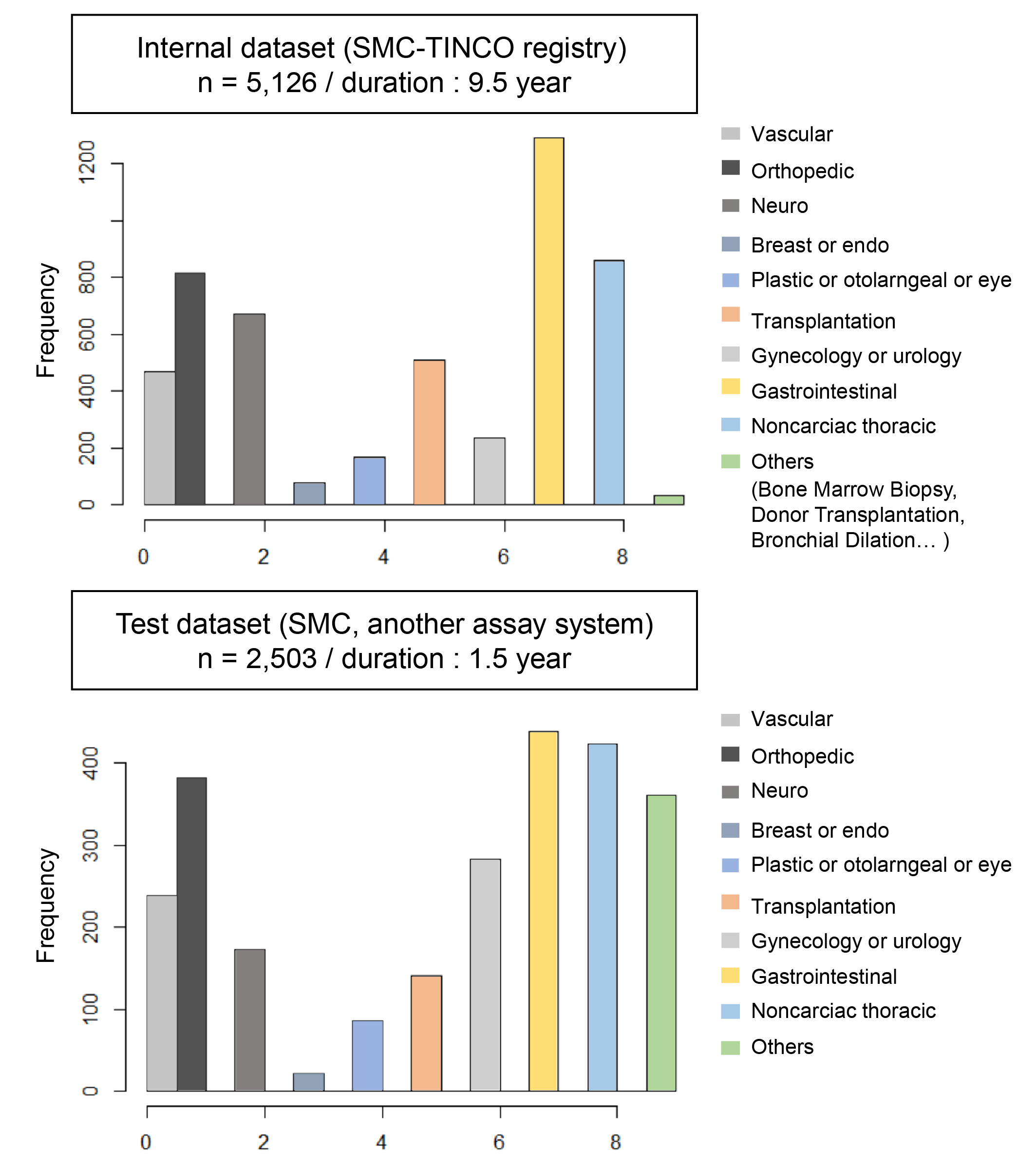

Supplement: Multimedia Appendix 5 [file medinform_v9i10e32771_app5.docx]
