# Supplementary material for: Predictability of Mortality in Patients With Myocardial Injury After Noncardiac Surgery Based on Perioperative Factors via Machine Learning: Retrospective Study
Source: JMIR Med Inform. 2021 Oct 14;9(10):e32771. doi: 10.2196/32771 (PMC8554678; doi:10.2196/32771)
Supplement: Multimedia Appendix 6 [file medinform_v9i10e32771_app6.docx]

**Multimedia Appendix 6.** Surgery type and mortality.

| **Surgery type** | **Internal dataset** | | | **Test dataset** | | |
| --- | --- | --- | --- | --- | --- | --- |
|  | **Patients number (%)** | **30-day mortality (%)** | **1-year mortality (%)** | **Patients number (%)** | **30-day mortality (%)** | **1-year mortality (%)** |
| Vascular | 468 (9.1) | 30 (4.8) | 46 (4.4) | 238 (9.5) | 2 (1.3) | 5 (2.3) |
| Orthopedic | 817 (15.9) | 52 (8.3) | 90 (8.7) | 379 (15.1) | 9 (5.9) | 18 (8.4) |
| Neurosurgery | 671 (13.1) | 128 (20.5) | 179 (17.2) | 173 (6.9) | 26 (17.0) | 32 (15.0) |
| Breast, endocrine | 77 (1.5) | 20 (3.2) | 32 (3.1) | 21 (0.8) | 0 (0.0) | 0 (0.0) |
| Plastic, ENT^a^, eye | 169 (3.3) | 27 (4.3) | 52 (5.2) | 85 (3.4) | 9 (5.9) | 13 (6.1) |
| Transplantation | 507 (9.9) | 49 (7.8) | 71 (6.8) | 138 (5.5) | 2 (1.3) | 2 (0.9) |
| OBGY^b^, Urology | 234 (4.6) | 30 (4.8) | 56 (5.4) | 278 (11.1) | 9 (5.9) | 17 (7.9) |
| Gastrointestinal | 1293 (25.2) | 171 (27.4) | 297 (28.6) | 439 (17.5) | 27 (17.6) | 37 (17.3) |
| Thoracic | 860 (16.8) | 114 (18.2) | 207 (19.9) | 415 (16.6) | 21 (13.7) | 28 (13.1) |
| Other | 30 (0.6) | 4 (0.6) | 10 (1.0) | 337 (13.5) | 48 (31.4) | 62 (29.0) |
| Total | 5,126 (100.0) | 625 (100.0) | 1,040 (100.0) | 2,503 (100.0) | 153 (100.0) | 218 (100.0) |

^a^ENT: Otolaryngology (Ear, Nose, Throat), ^b^OBGY: Obstetrics and Gynecology
