# Supplementary material for: Predictability of Mortality in Patients With Myocardial Injury After Noncardiac Surgery Based on Perioperative Factors via Machine Learning: Retrospective Study
Source: JMIR Med Inform. 2021 Oct 14;9(10):e32771. doi: 10.2196/32771 (PMC8554678; doi:10.2196/32771)
Supplement: Multimedia Appendix 7 [file medinform_v9i10e32771_app7.docx]

**Multimedia Appendix 7.** The optimal parameters of the machine learning algorithm.

| **Model** | **Hyperparameter** | **Full name** | **Values** |
| --- | --- | --- | --- |
| cart | cp | complexity parameter | 0.0005 |
| lda | - | - | - |
| svm | sigma | sigma | 0.005 |
|  | c | cost | 2.4 |
| knn | k | neighbors | 55 |
| rf | mtry | randomly selected predictors | 4 |
| glmnet | alpha | mixing percentage | 0.1 |
|  | lambda | regularization parameter | 0.001 |
| gbm | n.trees | boosting iterations | 80 |
|  | interaction.depth | max tree depth | 9 |
|  | shrinkage | learning rate /step-size reduction | 0.1 |
|  | n.minobsinnode | minimum observation in terminal node | 20 |
| xgboost | nrounds | number of iterations | 250 |
|  | max_depth | max tree depth | 4 |
|  | eta | learning rate | 0.055 |
|  | gamma | residual deviance | 0 |
|  | colsample_bytree | feature fraction | 1 |
|  | min_child_weight | minimum child weight | 1 |
|  | subsample | bagging fraction | 1 |
