# Supplementary material for: Predictability of Mortality in Patients With Myocardial Injury After Noncardiac Surgery Based on Perioperative Factors via Machine Learning: Retrospective Study
Source: JMIR Med Inform. 2021 Oct 14;9(10):e32771. doi: 10.2196/32771 (PMC8554678; doi:10.2196/32771)

**Multimedia Appendix 8.** AUROC and AUPRC plots of each model ((a) kNN, (b) CART, (c) LDA, (d) SVM, (e) GLMNET, (f) GBM, (g) RF, and (h) XGB) in predicting 30-days mortality.


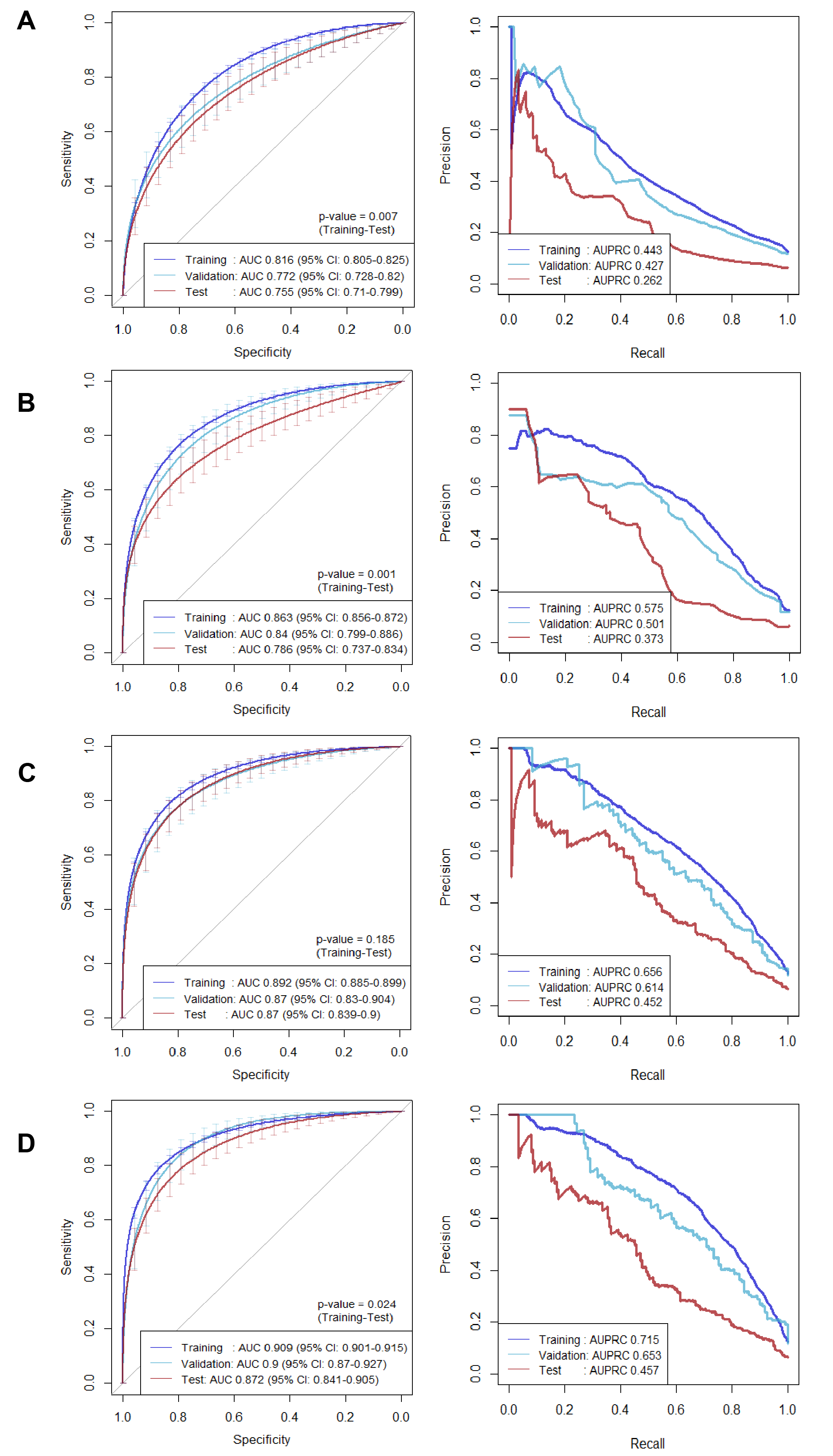


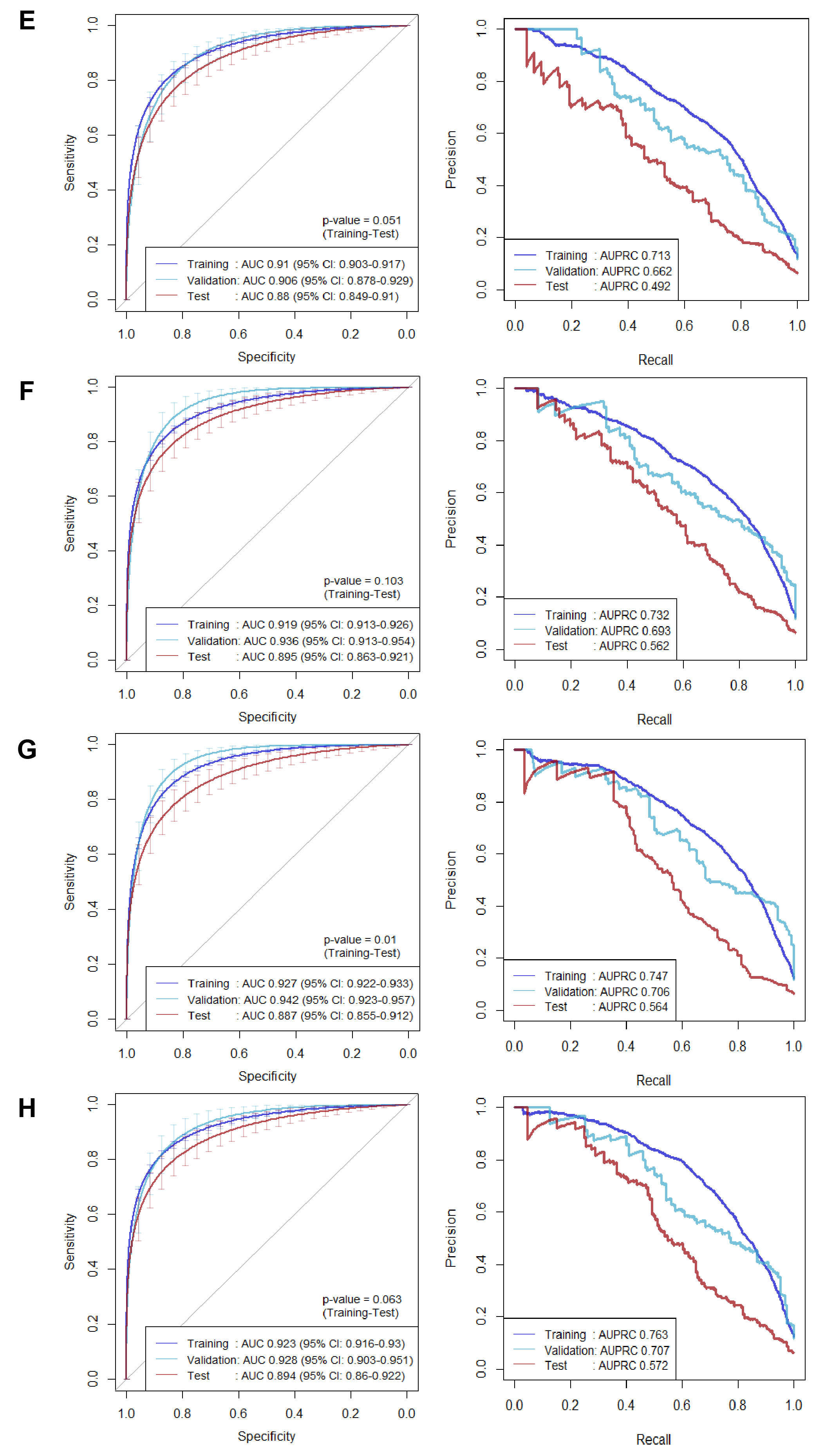

Supplement: Multimedia Appendix 8 [file medinform_v9i10e32771_app8.docx]
