# Supplementary material for: Predictability of Mortality in Patients With Myocardial Injury After Noncardiac Surgery Based on Perioperative Factors via Machine Learning: Retrospective Study
Source: JMIR Med Inform. 2021 Oct 14;9(10):e32771. doi: 10.2196/32771 (PMC8554678; doi:10.2196/32771)
Supplement: Multimedia Appendix 9 [file medinform_v9i10e32771_app9.docx]

**Multimedia Appendix 9**. Performance indexes of machine learning models predicting 30-day mortality of patients with MINS^a^.

|  | **CART^d^** | | | **LDA^e^** | | | **SVM^f^** | | | **kNN^g^** | | |
| --- | --- | --- | --- | --- | --- | --- | --- | --- | --- | --- | --- | --- |
|  | **Train** | **Internal** | **Test** | **Train** | **Internal** | **Test** | **Train** | **Internal** | **Test** | **Train** | **Internal** | **Test** |
| Accuracy | 0.899 | 0.903 | 0.939 | 0.911 | 0.912 | 0.946 | 0.921 | 0.914 | 0.948 | 0.889 | 0.895 | 0.939 |
| 95% CI lower of accuracy | 0.895 | 0.884 | 0.929 | 0.907 | 0.893 | 0.936 | 0.917 | 0.895 | 0.939 | 0.884 | 0.874 | 0.929 |
| 95% CI upper of accuracy | 0.903 | 0.921 | 0.948 | 0.915 | 0.929 | 0.955 | 0.925 | 0.931 | 0.957 | 0.893 | 0.913 | 0.948 |
| No information rate (NIR) | 0.877 | 0.883 | 0.939 | 0.877 | 0.883 | 0.939 | 0.877 | 0.883 | 0.939 | 0.877 | 0.883 | 0.939 |
| P-value [Accuracy > NIR] | 0.000 | 0.021 | 0.488 | 0.000 | 0.002 | 0.070 | 0.000 | 0.001 | 0.023 | 0.000 | 0.131 | 0.488 |
| Sensitivity | 0.503 | 0.475 | 0.359 | 0.469 | 0.392 | 0.418 | 0.514 | 0.450 | 0.353 | 0.129 | 0.133 | 0.118 |
| Specificity | 0.955 | 0.960 | 0.977 | 0.973 | 0.981 | 0.980 | 0.978 | 0.976 | 0.987 | 0.995 | 0.996 | 0.993 |
| AUROC^b^ | 0.863 | 0.840 | 0.786 | 0.892 | 0.870 | 0.870 | 0.909 | 0.900 | 0.872 | 0.816 | 0.772 | 0.755 |
| Positive predictive value | 0.610 | 0.613 | 0.505 | 0.708 | 0.734 | 0.582 | 0.770 | 0.711 | 0.643 | 0.789 | 0.800 | 0.514 |
| Negative predictive value | 0.932 | 0.932 | 0.959 | 0.929 | 0.924 | 0.963 | 0.935 | 0.930 | 0.959 | 0.891 | 0.897 | 0.945 |
| Precision | 0.610 | 0.613 | 0.505 | 0.708 | 0.734 | 0.582 | 0.770 | 0.711 | 0.643 | 0.789 | 0.800 | 0.514 |
| Recall | 0.503 | 0.475 | 0.359 | 0.469 | 0.392 | 0.418 | 0.514 | 0.450 | 0.353 | 0.129 | 0.133 | 0.118 |
| F1 score | 0.551 | 0.535 | 0.420 | 0.565 | 0.511 | 0.487 | 0.616 | 0.551 | 0.456 | 0.222 | 0.229 | 0.191 |
| AUPRC^c^ | 0.575 | 0.501 | 0.373 | 0.656 | 0.614 | 0.452 | 0.715 | 0.653 | 0.457 | 0.443 | 0.427 | 0.262 |
| Prevalence | 0.123 | 0.117 | 0.061 | 0.123 | 0.117 | 0.061 | 0.123 | 0.117 | 0.061 | 0.123 | 0.117 | 0.061 |
| Detection rate | 0.062 | 0.056 | 0.022 | 0.058 | 0.046 | 0.026 | 0.063 | 0.053 | 0.022 | 0.016 | 0.016 | 0.007 |
| Detection prevalence | 0.101 | 0.091 | 0.044 | 0.082 | 0.062 | 0.044 | 0.082 | 0.074 | 0.034 | 0.020 | 0.020 | 0.014 |
| Balanced accuracy | 0.729 | 0.718 | 0.668 | 0.721 | 0.686 | 0.699 | 0.746 | 0.713 | 0.670 | 0.562 | 0.564 | 0.555 |

**Multimedia Appendix 9.** (Continued)

|  | **RF^h^** | | | **GLMNET^i^** | | | **GBM^j^** | | | **XGB^k^** | | |
| --- | --- | --- | --- | --- | --- | --- | --- | --- | --- | --- | --- | --- |
|  | **Train** | **Internal** | **Test** | **Train** | **Internal** | **Test** | **Train** | **Internal** | **Test** | **Train** | **Internal** | **Test** |
| Accuracy | 0.920 | 0.916 | 0.950 | 0.919 | 0.913 | 0.952 | 0.922 | 0.918 | 0.952 | 0.931 | 0.922 | 0.954 |
| 95% CI lower of accuracy | 0.916 | 0.897 | 0.940 | 0.915 | 0.894 | 0.943 | 0.918 | 0.900 | 0.943 | 0.928 | 0.904 | 0.945 |
| 95% CI lower of accuracy | 0.923 | 0.932 | 0.958 | 0.922 | 0.930 | 0.960 | 0.925 | 0.934 | 0.960 | 0.935 | 0.938 | 0.962 |
| No information rate (NIR) | 0.877 | 0.883 | 0.939 | 0.877 | 0.883 | 0.939 | 0.877 | 0.883 | 0.939 | 0.877 | 0.883 | 0.939 |
| P-value [Acc > NIR] | 0.000 | 0.000 | 0.012 | 0.000 | 0.001 | 0.003 | 0.000 | 0.000 | 0.003 | 0.000 | 0.000 | 0.001 |
| Sensitivity | 0.398 | 0.325 | 0.196 | 0.500 | 0.408 | 0.359 | 0.544 | 0.425 | 0.340 | 0.588 | 0.475 | 0.314 |
| Specificity | 0.993 | 0.994 | 0.999 | 0.977 | 0.980 | 0.990 | 0.975 | 0.983 | 0.991 | 0.979 | 0.981 | 0.996 |
| AUROC^b^ | 0.927 | 0.942 | 0.887 | 0.910 | 0.906 | 0.880 | 0.919 | 0.936 | 0.895 | 0.923 | 0.928 | 0.894 |
| Positive predictive value | 0.886 | 0.886 | 0.909 | 0.755 | 0.731 | 0.705 | 0.752 | 0.773 | 0.722 | 0.801 | 0.770 | 0.828 |
| Negative predictive value | 0.922 | 0.917 | 0.950 | 0.933 | 0.926 | 0.960 | 0.938 | 0.928 | 0.958 | 0.944 | 0.934 | 0.957 |
| Precision | 0.886 | 0.886 | 0.909 | 0.755 | 0.731 | 0.705 | 0.752 | 0.773 | 0.722 | 0.801 | 0.770 | 0.828 |
| Recall | 0.398 | 0.325 | 0.196 | 0.500 | 0.408 | 0.359 | 0.544 | 0.425 | 0.340 | 0.588 | 0.475 | 0.314 |
| F1 score | 0.549 | 0.476 | 0.323 | 0.602 | 0.524 | 0.476 | 0.631 | 0.548 | 0.462 | 0.678 | 0.588 | 0.455 |
| AUPRC^c^ | 0.747 | 0.706 | 0.564 | 0.713 | 0.662 | 0.492 | 0.732 | 0.693 | 0.562 | 0.763 | 0.707 | 0.572 |
| Prevalence | 0.123 | 0.117 | 0.061 | 0.123 | 0.117 | 0.061 | 0.123 | 0.117 | 0.061 | 0.123 | 0.117 | 0.061 |
| Detection rate | 0.049 | 0.038 | 0.012 | 0.062 | 0.048 | 0.022 | 0.067 | 0.050 | 0.021 | 0.072 | 0.056 | 0.019 |
| Detection prevalence | 0.055 | 0.043 | 0.013 | 0.082 | 0.065 | 0.031 | 0.089 | 0.064 | 0.029 | 0.090 | 0.072 | 0.023 |
| Balanced accuracy | 0.695 | 0.660 | 0.597 | 0.739 | 0.694 | 0.675 | 0.759 | 0.704 | 0.666 | 0.784 | 0.728 | 0.655 |

^a^MINS: myocardial injury after noncardiac surgery, ^b^AUROC: Area Under the Receiver Operating Characteristic, ^c^AUPRC: Area Under the Precision Recall Curve, ^d^CART: Classification and Regression Trees, ^e^LDA: Linear Discriminant Analysis, ^f^SVM: Support Vector Machines, ^g^kNN: k-nearest Neighbors, ^h^RF: Random Forests, ^i^GLMNET: Lasso/ridge/elastic Net, ^j^GBM: Generalized Boosted Regression Model, ^k^XGB: Extreme Gradient Boosting.
