# Supplementary material for: Predictability of Mortality in Patients With Myocardial Injury After Noncardiac Surgery Based on Perioperative Factors via Machine Learning: Retrospective Study
Source: JMIR Med Inform. 2021 Oct 14;9(10):e32771. doi: 10.2196/32771 (PMC8554678; doi:10.2196/32771)

**Multimedia Appendix 11.** SHAP dependence plots for top 10 features of 30-day mortality prediction model; (a) antiplatelet prescription at discharge, (b) C-reactive protein level at discharge, (c) insulin prescription at discharge, (d) beta blocker, (e) peak cardiac troponin level (ng/L), (f) statin prescription at discharge.


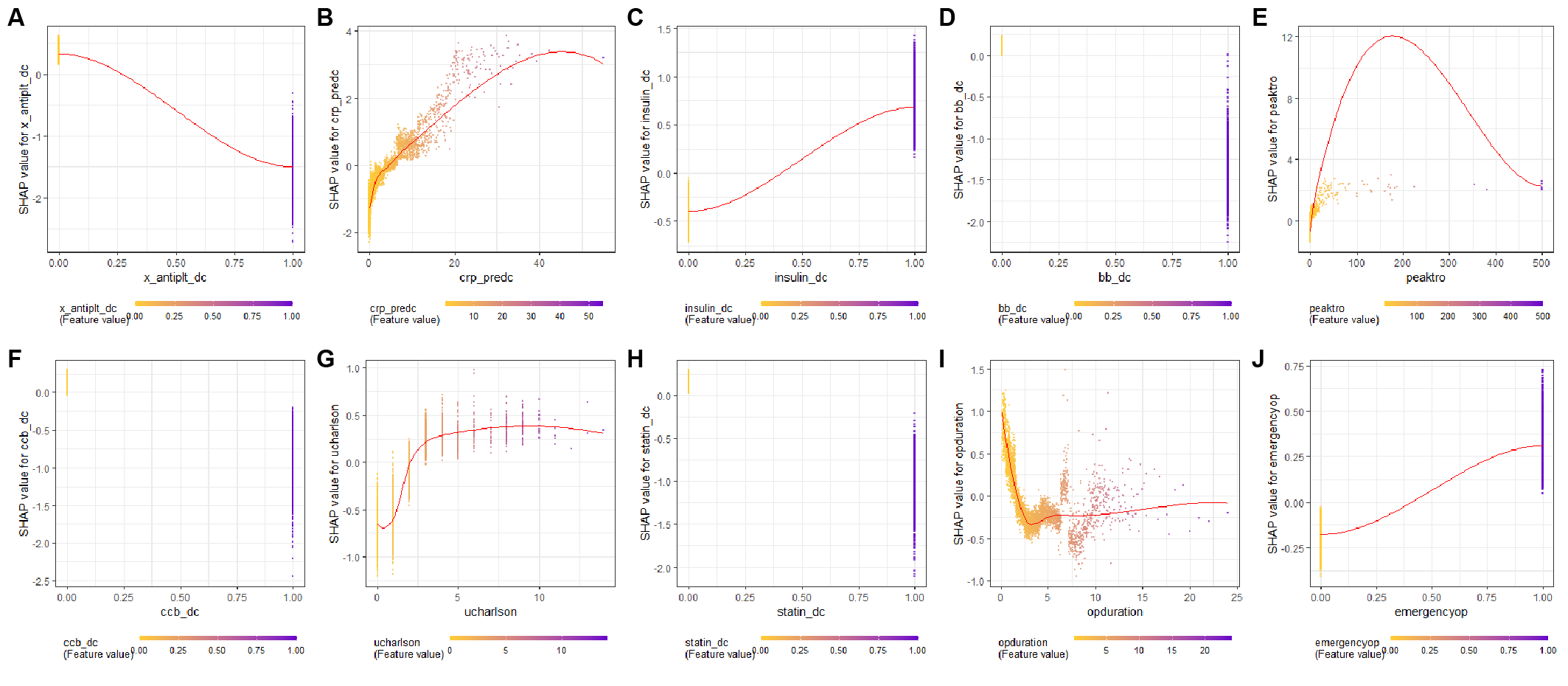

Supplement: Multimedia Appendix 11 [file medinform_v9i10e32771_app11.docx]
