# Supplementary material for: Predictability of Mortality in Patients With Myocardial Injury After Noncardiac Surgery Based on Perioperative Factors via Machine Learning: Retrospective Study
Source: JMIR Med Inform. 2021 Oct 14;9(10):e32771. doi: 10.2196/32771 (PMC8554678; doi:10.2196/32771)

**Multimedia Appendix 12.** Recursive Feature Elimination (RFE) graph based on accuracy (upper). The RFE algorithms recommended the use of 28 variables (filled blue dot); c-reactive protein level at discharge, insulin, antiplatelet, peak cardiac troponin level (ng/L), calcium channel blocker, emergency operation, operation duration (hours), stain, beta blocker, operation type, ucharlson score, renin angiotensin aldosterone system inhibitor, charlson score, metformin, active cancer, direct oral anticoagulant, ESC/ESA surgical high risk, diltiazem, diabetes, age, ga, dialysis, hypertension, CAGB history, coronary artery disease, chronic renal failure, percutaneous intervention history, and coronary revascularization. ESC: European Society of Cardiology, ESA: European Society of Anaesthesiology, CABG: Coronary Artery Bypass Grafting


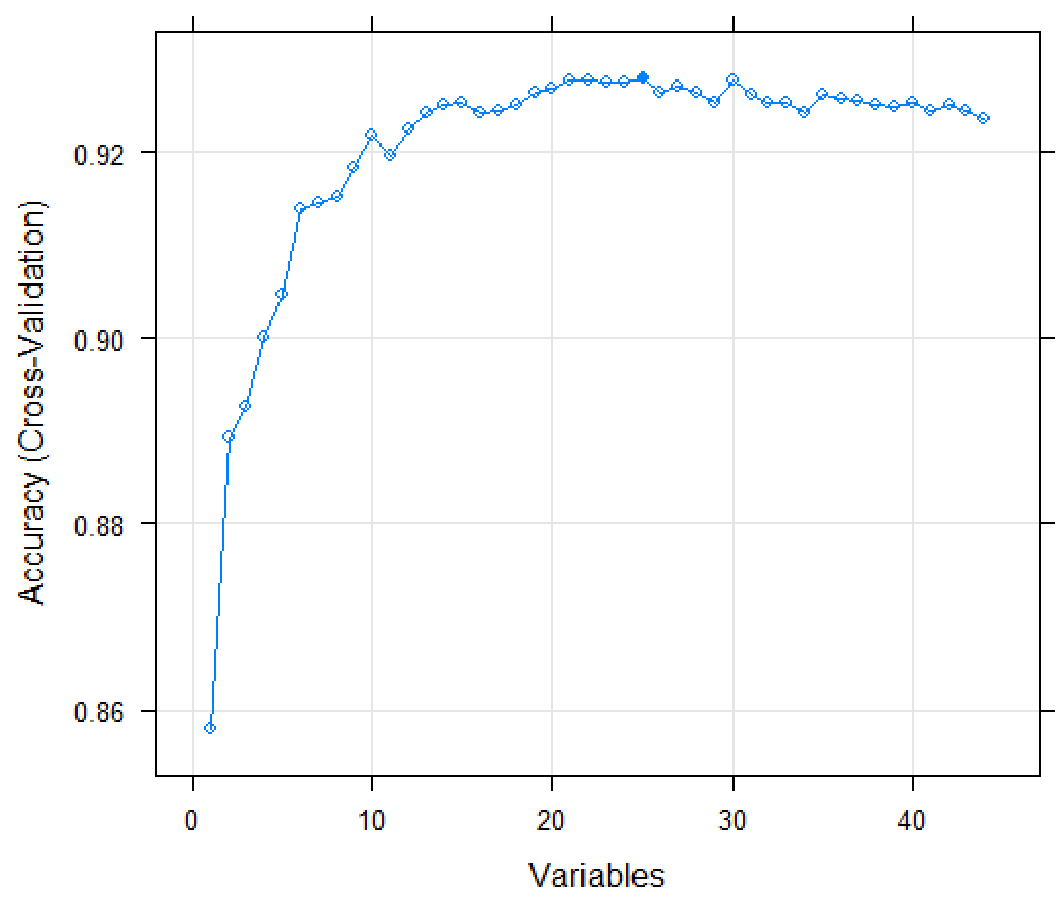

Supplement: Multimedia Appendix 12 [file medinform_v9i10e32771_app12.docx]
