# Supplementary material for: Predictability of Mortality in Patients With Myocardial Injury After Noncardiac Surgery Based on Perioperative Factors via Machine Learning: Retrospective Study
Source: JMIR Med Inform. 2021 Oct 14;9(10):e32771. doi: 10.2196/32771 (PMC8554678; doi:10.2196/32771)
Supplement: Multimedia Appendix 13 [file medinform_v9i10e32771_app13.docx]

**Multimedia Appendix 13**. Performance indexes of XGB models predicting 30-day mortality of MINS^a^ patients with top 28, top 10, chosen 10, and top 5 variables.

|  | **Top 28** | | | **Top 10** | | | **Chosen 10** | | | **Top 5** | | |
| --- | --- | --- | --- | --- | --- | --- | --- | --- | --- | --- | --- | --- |
|  | **Train** | **Internal** | **Test** | **Train** | **Internal** | **Test** | **Train** | **Internal** | **Test** | **Train** | **Internal** | **Test** |
| Accuracy | 0.926 | 0.917 | 0.955 | 0.920 | 0.922 | 0.955 | 0.916 | 0.908 | 0.948 | 0.907 | 0.911 | 0.950 |
| 95% CI lower of accuracy | 0.922 | 0.898 | 0.946 | 0.916 | 0.904 | 0.946 | 0.912 | 0.889 | 0.939 | 0.903 | 0.892 | 0.940 |
| 95% CI upper of accuracy | 0.930 | 0.933 | 0.963 | 0.923 | 0.938 | 0.963 | 0.919 | 0.925 | 0.956 | 0.911 | 0.928 | 0.958 |
| No information rate (NIR) | 0.877 | 0.883 | 0.939 | 0.877 | 0.883 | 0.939 | 0.877 | 0.883 | 0.939 | 0.877 | 0.883 | 0.939 |
| P-value [Accuracy > NIR] | 0.000 | 0.000 | 0.000 | 0.000 | 0.000 | 0.000 | 0.000 | 0.005 | 0.028 | 0.000 | 0.002 | 0.012 |
| Sensitivity | 0.562 | 0.442 | 0.340 | 0.522 | 0.475 | 0.392 | 0.488 | 0.392 | 0.359 | 0.384 | 0.333 | 0.235 |
| Specificity | 0.977 | 0.980 | 0.995 | 0.976 | 0.981 | 0.991 | 0.976 | 0.977 | 0.986 | 0.981 | 0.988 | 0.996 |
| AUROC^b^ | 0.925 | 0.934 | 0.908 | 0.911 | 0.914 | 0.904 | 0.894 | 0.912 | 0.895 | 0.890 | 0.902 | 0.885 |
| Positive predictive value | 0.777 | 0.746 | 0.825 | 0.751 | 0.770 | 0.750 | 0.738 | 0.691 | 0.632 | 0.738 | 0.784 | 0.800 |
| Negative predictive value | 0.941 | 0.930 | 0.959 | 0.936 | 0.934 | 0.962 | 0.931 | 0.924 | 0.959 | 0.919 | 0.918 | 0.952 |
| Precision | 0.777 | 0.746 | 0.825 | 0.751 | 0.770 | 0.750 | 0.738 | 0.691 | 0.632 | 0.738 | 0.784 | 0.800 |
| Recall | 0.562 | 0.442 | 0.340 | 0.522 | 0.475 | 0.392 | 0.488 | 0.392 | 0.359 | 0.384 | 0.333 | 0.235 |
| F1 score | 0.652 | 0.555 | 0.481 | 0.616 | 0.588 | 0.515 | 0.587 | 0.500 | 0.458 | 0.505 | 0.468 | 0.364 |
| AUPRC^c^ | 0.754 | 0.704 | 0.582 | 0.708 | 0.695 | 0.587 | 0.672 | 0.690 | 0.489 | 0.640 | 0.627 | 0.500 |
| Prevalence | 0.123 | 0.117 | 0.061 | 0.123 | 0.117 | 0.061 | 0.123 | 0.117 | 0.061 | 0.123 | 0.117 | 0.061 |
| Detection rate | 0.069 | 0.052 | 0.021 | 0.064 | 0.056 | 0.024 | 0.060 | 0.046 | 0.022 | 0.047 | 0.039 | 0.014 |
| Detection prevalence | 0.089 | 0.069 | 0.025 | 0.086 | 0.072 | 0.032 | 0.081 | 0.066 | 0.035 | 0.064 | 0.050 | 0.018 |
| Balanced accuracy | 0.769 | 0.711 | 0.668 | 0.749 | 0.728 | 0.692 | 0.732 | 0.684 | 0.673 | 0.682 | 0.661 | 0.616 |

^a^MINS: myocardial injury after noncardiac surgery, ^b^AUROC: Area Under the Receiver Operating Characteristic, ^c^AUPRC: Area Under the Precision Recall Curve
