# Supplementary material for: Predictability of Mortality in Patients With Myocardial Injury After Noncardiac Surgery Based on Perioperative Factors via Machine Learning: Retrospective Study
Source: JMIR Med Inform. 2021 Oct 14;9(10):e32771. doi: 10.2196/32771 (PMC8554678; doi:10.2196/32771)

**Multimedia Appendix 14**. AUROC (left) and AUPRC (right) plots of each model predicting 30-days mortality with (a) 28, (b) 10, (c) chosen 10, (d) 5 variables.


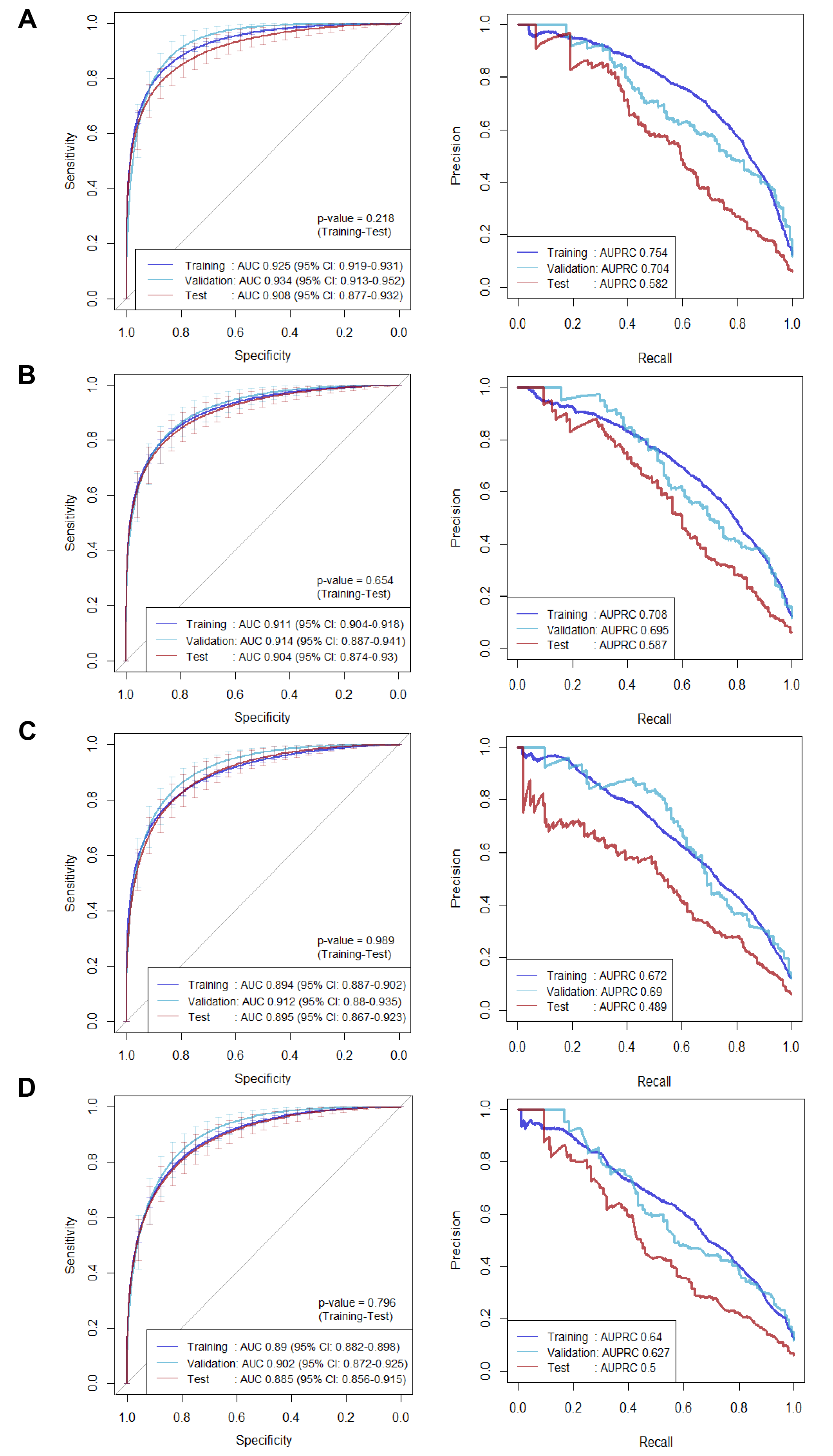

Supplement: Multimedia Appendix 14 [file medinform_v9i10e32771_app14.docx]
