# Supplementary material for: Predictability of Mortality in Patients With Myocardial Injury After Noncardiac Surgery Based on Perioperative Factors via Machine Learning: Retrospective Study
Source: JMIR Med Inform. 2021 Oct 14;9(10):e32771. doi: 10.2196/32771 (PMC8554678; doi:10.2196/32771)
Supplement: Multimedia Appendix 16 [file medinform_v9i10e32771_app16.docx]

**Multimedia Appendix 16**. Performance indexes of XGB^a^ model predicting one-year mortality of patients with MINS^b^.

|  | **XGB^a^** | | |
| --- | --- | --- | --- |
|  | **Train** | **Internal** | **Test** |
| Accuracy | 0.931 | 0.922 | 0.954 |
| 95% CI lower of accuracy | 0.928 | 0.904 | 0.945 |
| 95% CI upper of accuracy | 0.935 | 0.938 | 0.962 |
| No information rate (NIR) | 0.877 | 0.883 | 0.939 |
| P-value [Acc > NIR] | 0.000 | 0.000 | 0.001 |
| Sensitivity | 0.588 | 0.475 | 0.314 |
| Specificity | 0.979 | 0.981 | 0.996 |
| AUROC^c^ | 0.857 | 0.859 | 0.794 |
| Positive predictive value | 0.801 | 0.770 | 0.828 |
| Negative predictive value | 0.944 | 0.934 | 0.957 |
| Precision | 0.801 | 0.770 | 0.828 |
| Recall | 0.588 | 0.475 | 0.314 |
| F1 score | 0.678 | 0.588 | 0.455 |
| AUPRC^d^ | 0.685 | 0.663 | 0.458 |
| Prevalence | 0.123 | 0.117 | 0.061 |
| Detection rate | 0.072 | 0.056 | 0.019 |
| Detection prevalence | 0.090 | 0.072 | 0.023 |
| Balanced accuracy | 0.784 | 0.728 | 0.655 |

^a^XGB: Extreme Gradient Boosting, ^b^MINS: myocardial injury after noncardiac surgery, ^c^AUROC: Area Under the Receiver Operating Characteristic, ^d^AUPRC: Area Under the Precision Recall Curve
