# Supplementary material for: Predictability of Mortality in Patients With Myocardial Injury After Noncardiac Surgery Based on Perioperative Factors via Machine Learning: Retrospective Study
Source: JMIR Med Inform. 2021 Oct 14;9(10):e32771. doi: 10.2196/32771 (PMC8554678; doi:10.2196/32771)
Supplement: Multimedia Appendix 19 [file medinform_v9i10e32771_app19.docx]

**Multimedia Appendix 19.** Optimized threshold and final performance of models.

| **Model** | **Threshold** | **Accuracy** | **Balanced accuracy** | **F1 score** |
| --- | --- | --- | --- | --- |
| Top 28 | 0.43 | 0.956 | 0.680 | 0.502 |
| Top 10 | 0.41 | 0.955 | 0.716 | 0.546 |
| Chosen 10 | 0.45 | 0.947 | 0.669 | 0.450 |
| Top 5 | 0.32 | 0.948 | 0.688 | 0.480 |
