# Supplementary material for: Predictability of Mortality in Patients With Myocardial Injury After Noncardiac Surgery Based on Perioperative Factors via Machine Learning: Retrospective Study
Source: JMIR Med Inform. 2021 Oct 14;9(10):e32771. doi: 10.2196/32771 (PMC8554678; doi:10.2196/32771)

**Multimedia Appendix 20.** Comparison of the SHAP value with the Charlson comorbidity index score (up) and updated Charlson comorbidity index score (down). The original Charlson score is proportional to an increase in SHAP value. However, the updated scoring system shows an early warning signal with SHAP value, but a higher score did not guarantee a higher SHAP value.


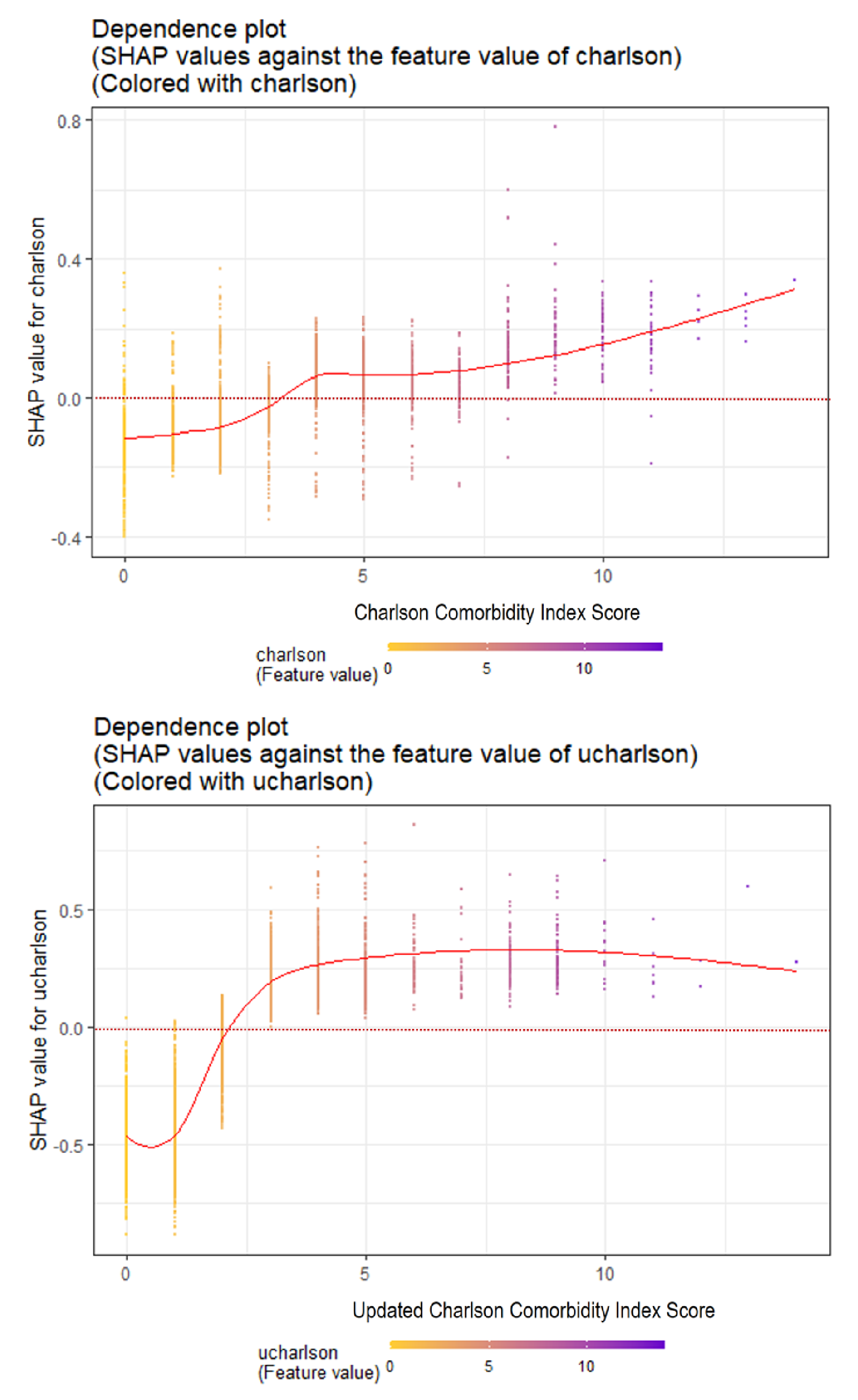

Supplement: Multimedia Appendix 20 [file medinform_v9i10e32771_app20.docx]
